# Translocation of the papillomavirus L2/vDNA complex across the limiting membrane requires the onset of mitosis

**DOI:** 10.1371/journal.ppat.1006200

**Published:** 2017-05-02

**Authors:** Christine M. Calton, Matthew P. Bronnimann, Ariana R. Manson, Shuaizhi Li, Janice A. Chapman, Marcela Suarez-Berumen, Tatum R. Williamson, Sudheer K. Molugu, Ricardo A. Bernal, Samuel K. Campos

**Affiliations:** 1 BIO5 Institute, University of Arizona, Tucson, Arizona, United States of America; 2 Department of Immunobiology, University of Arizona, Tucson, Arizona, United States of America; 3 Department of Molecular & Cellular Biology, University of Arizona, Tucson, Arizona, United States of America; 4 Department of Cellular & Molecular Medicine, University of Arizona, Tucson, Arizona, United States of America; 5 Department of Chemistry, University of Texas at El Paso, El Paso, Texas, United States of America; 6 Cancer Biology Graduate Interdisciplinary Program, University of Arizona, Tucson, Arizona, United States of America; Penn State University School of Medicine, UNITED STATES

## Abstract

The human papillomavirus type 16 (HPV16) L2 protein acts as a chaperone to ensure that the viral genome (vDNA) traffics from endosomes to the *trans*-Golgi network (TGN) and eventually the nucleus, where HPV replication occurs. En route to the nucleus, the L2/vDNA complex must translocate across limiting intracellular membranes. The details of this critical process remain poorly characterized. We have developed a system based on subcellular compartmentalization of the enzyme BirA and its cognate substrate to detect membrane translocation of L2-BirA from incoming virions. We find that L2 translocation requires transport to the TGN and is strictly dependent on entry into mitosis, coinciding with mitotic entry in synchronized cells. Cell cycle arrest causes retention of L2/vDNA at the TGN; only release and progression past G2/M enables translocation across the limiting membrane and subsequent infection. Microscopy of EdU-labeled vDNA reveals a rapid and dramatic shift in vDNA localization during early mitosis. At late G2/early prophase vDNA egresses from the TGN to a pericentriolar location, accumulating there through prometaphase where it begins to associate with condensed chromosomes. By metaphase and throughout anaphase the vDNA is seen bound to the mitotic chromosomes, ensuring distribution into both daughter nuclei. Mutations in a newly defined chromatin binding region of L2 potently blocked translocation, suggesting that translocation is dependent on chromatin binding during prometaphase. This represents the first time a virus has been shown to functionally couple the penetration of limiting membranes to cellular mitosis, explaining in part the tropism of HPV for mitotic basal keratinocytes.

## Author summary

Human papillomaviruses (HPVs) are the most prevalent sexually transmitted infections and are responsible for about 5% of total human cancers worldwide. These small DNA viruses must enter cells and deliver their genomes to the host cell nucleus to successfully establish an infection. A major barrier that HPVs and other non-enveloped viruses must overcome is the limiting membrane—the host cellular membrane through which the viral genome must be transported en route to the nucleus. Here we have developed a novel enzyme-dependent assay to study what is called penetration or translocation of the limiting membrane by the HPV16 minor capsid protein L2. We find that translocation is strictly dependent on host cell division, specifically progression into mitosis, and occurs post *trans*-Golgi-network localization. After exiting the *trans*-Golgi network the viral genomes are seen decorating the segregating mitotic chromosomes, to ensure the equal partitioning into and infection of the daughter cells. The remarkable timing with, and absolute dependence of, this event on mitosis likely dictates the natural tropism of these viruses for the replicating basal keratinocytes of differentiating epithelial tissues. Further work will be necessary to fully understand the molecular mechanisms of this extraordinary mitosis-dependent phenomenon.

## Introduction

Human papillomaviruses (HPVs) are small, double-stranded DNA viruses that infect the basal keratinocytes of differentiating epidermal or mucosal epithelium. Most HPV infections are asymptomatic or cause benign lesions. However, several HPV types, termed high-risk HPVs, are associated with essentially all cases of cervical cancer, as well as a significant number of anogenital and oropharyngeal cancers [1]. HPV16 is the most common high-risk type and accounts for over 60% of cervical cancer cases. Combined, HPVs are responsible for approximately 5% of all human cancers [2].

The HPV16 capsid is a non-enveloped 55 nm icosahedral structure that consists of 72 pentamers of the major capsid protein L1. Within the particle are approximately 20–40 copies of the minor capsid protein L2, in complex with a circularized, 8 kb chromatinized genome [3, 4]. Despite its designation as a minor capsid protein, L2 plays important roles in capsid assembly and genome encapsidation [5–7], and is essential for establishing infection within the cell [8].

Initial attachment of HPV16 is mediated via electrostatic interactions with heparan sulfate proteoglycans (HSPGs) on either the cell-surface or extracellular matrix (ECM) or through transient binding to ECM resident laminin 332 [9–14]. Binding to HSPGs induces conformational changes in the capsid that are detectable by the exposure of masked epitopes in both L1 and L2 and facilitate cleavage of these capsid proteins by the host proteases kallikrein-8 and furin respectively [10, 15, 16]. These conformational changes are also believed to mediate transfer of the virion to an entry receptor complex that allows endocytosis in an actin-dependent manner [17, 18].

Once inside the cell, HPV traffics through the endosomal pathway, where acidification promotes further capsid disassembly and degradation [15, 19–21]. Host cyclophilins facilitate release of the L2/vDNA complex from the remnants of the L1 capsid structure [22], thereby enabling transport to the *trans*-Golgi network (TGN) and possibly other distal compartments via retrograde trafficking pathways [23–26]. Retrograde transport to the TGN is dependent upon the activity of furin and γ-secretase [23, 25], as well as interactions between L2 and cytosolic host trafficking factors including sorting nexin 17 (SNX17), sorting nexin 27 (SNX27), and the retromer complex [27–30]. Based on immunofluorescence microscopy and colocalization studies, TGN/Golgi localization of L2/vDNA has been proposed to be necessary for translocation to occur, but the details of this translocation event remain poorly defined [23, 31]. After exiting the TGN, the L2/vDNA complex traffics to the nucleus where it associates with PML bodies and initiates early viral transcription and replication [8]. This event is poorly defined but thought to require mitotic breakdown of the nuclear envelope, as cell cycle inhibitors block nuclear localization of L2/vDNA and productive infection [31–33]. Prior work has suggested that L2 can bind to mitotic chromatin as a means to ensure nuclear localization in the infected daughter cells [31]. Following up on this idea, the accompanying manuscript by the Schelhaas group defines a 147 residue (aa 188–334) chromatin binding region of HPV16 L2 that is necessary and sufficient for the binding of L2 to mitotic chromosomes [34]. Notably, the association of L2 with mitotic chromatin requires progression into prometaphase, and is likely indirect, involving an unknown host mitotic factor or factors.

How L2 and the viral genome make the leap from the TGN to the nucleus is unclear. A recent report presents evidence that L2/vDNA remains in a vesicular compartment after egress from the TGN during mitosis [35]. At some point on its journey to the nucleus, the L2/vDNA complex must physically penetrate or translocate across a limiting lipid membrane within the cell. A detailed understanding of this translocation has been hampered by a lack of suitable techniques that are sensitive enough to detect the event. In this report, we have developed a novel assay to monitor L2 translocation that depends on fusion of L2 to the bacterial biotin ligase BirA [36]. The assay is based on the compartmentalization of a specific BirA enzyme-substrate reaction. Virions encapsidating the L2-BirA fusion are used to infect host cells that stably express a cytosolically localized BirA substrate. Only when L2 translocates across the limiting membrane will the BirA encounter and biotinylate the substrate. Translocation is therefore directly correlated to substrate biotinylation, which can easily be detected by western blotting and neutravidin staining.

Using this simple but innovative assay, we show that inhibition of the cellular processes and factors that are essential for L2/vDNA trafficking to the TGN, including furin, endosome acidification, cyclophilins, and γ-secretase, potently block L2 translocation. Treatments that induce cell cycle arrest also inhibit L2 translocation and cause vDNA to accumulate at the TGN. We find that mitotic entry is sufficient for L2 translocation to occur and that translocation coincides with mitosis in synchronized cells. Furthermore, during the onset of mitosis vDNA moves rapidly from the TGN to a pericentriolar region before associating with mitotic host chromosomes during prometaphase, ensuring infection of both daughter cells. Finally, here and in the accompanying manuscript, we present evidence that translocation is blocked in three different mutants of the newly defined chromatin binding region of L2 [34], suggesting a functional link between the ability to bind chromatin and L2/vDNA translocation. Based on these findings we propose a model whereby chromatin binding of L2 in prometaphase is a necessary prerequisite for translocation of L2/vDNA across limiting membranes.

## Results

### Development of a BirA-based translocation system

**Fig 1:**
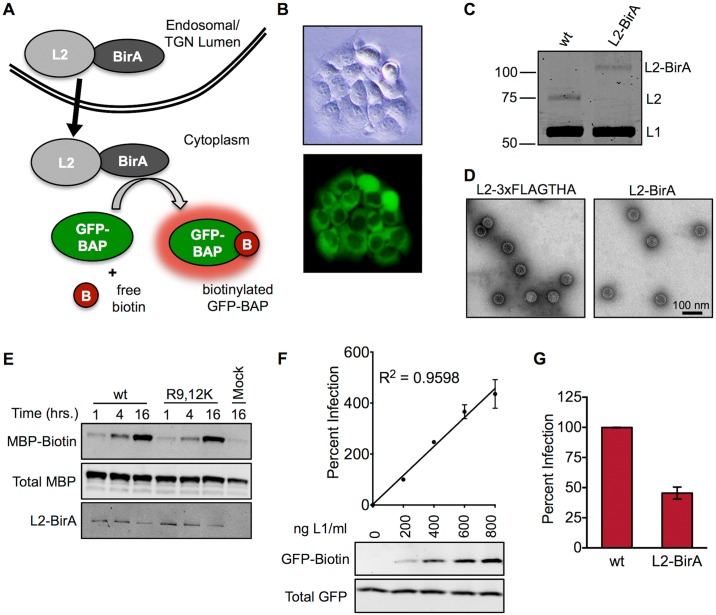
Development of the BirA/GFP-BAP assay for detecting membrane penetration by L2. **(A)** Schematic of the BirA translocation assay. L2-BirA is physically separated from its substrate (GFP-BAP) while in the lumen of endocytic vesicles. Following translocation across the limiting membrane, L2-BirA can biotinylate cytosolic GFP-BAP. **(B)** Brightfield and epifluorescent imaging of HaCaT GFP-BAP cells. **(C)** Coomassie staining of wt-L2 and L2-BirA PsV. Molecular weights are shown in kilodaltons. **(D)** Transmission electron microscopy imaging of L2-3xFLAGTHA or L2-BirA viral particles. Scale bar represents 100 nm. **(E)** *In vitro* biotinylation of reduced wt L2-BirA or R9,12K L2-BirA particles incubated with MBP-BAP, ATP, and biotin for the indicated times prior to processing by SDS-PAGE/Western blotting. **(F)** Titration of infectivity and translocation of L2-BirA in HaCaT GFP-BAP cells. Graph shows percent infectivity, relative to the 200 ng L1/ml concentration, which is set at 100%. GFP-biotin and total GFP were analysed by Western blotting with neutravidin and anti-GFP staining respectively. **(G)** Infectivity of wt and L2-BirA pseudovirions in HaCaT GFP-BAP cells at an equal MOI, expressed relative to wt, which is set at 100%. All infection values represent mean percent infection (±SEM, *n* = 2–3), normalized to GAPDH.

Prior efforts to study L2/vDNA translocation across the limiting membrane have relied heavily on confocal microscopy and colocalization of EdU-labeled vDNA with subcellular markers [23, 31, 37]. While informative, these methods are unsuitable for drawing definitive conclusions on the lumenal versus cytosolic state of the L2/vDNA complex. To detect egress of virion-derived L2 out of vesicular compartments, we developed a membrane translocation assay that utilizes an L2 fusion to the *Escherichia coli* biotin ligase BirA [36, 38] (Fig 1A). HaCaT keratinocytes were transfected with pCIP-NES-GFP-BAP to isolate a subclone that stably expresses cytosolically localized GFP fused to a 15 amino acid biotin acceptor peptide (HaCaT GFP-BAP cells, Fig 1B). BAP is an engineered BirA-specific substrate that cannot be biotinylated by holocarboxylase synthetase, the orthologous mammalian biotin ligase [39–41]. In this system, L2-BirA must traverse the limiting membrane to encounter cytosolic GFP-BAP. BirA-dependent biotinylation of GFP-BAP is therefore a direct readout of L2-BirA membrane translocation. Luciferase expressing HPV16 L2-BirA pseudovirions (PsV) were generated as described in *Materials and Methods*. L2-BirA particles incorporate L2 at levels similar to wild type (wt) particles (Fig 1C) and exhibit normal capsid morphology when examined by transmission electron microscopy (Fig 1D). *In vitro* biotin ligase reactions were performed with PsV containing wt L2 or the non-infectious R9,12K furin cleavage site mutant L2 [42]. Both were capable of biotinylating BAP-tagged maltose binding protein (Fig 1E), demonstrating that BirA retains activity in the context of an L2 fusion and that the purified PsV contain active BirA enzyme. Infection of HaCaT GFP-BAP cells with L2-BirA results in biotinylation of GFP-BAP and luciferase expression in a dose-dependent manner (Fig 1F). L2-BirA is less infectious than PsV lacking the large C-terminal BirA fusion (Fig 1G), and we have observed particle instability after prolonged storage at 4°C. It is therefore recommended that aliquots be stored at -80°C and that the concentration of virus be verified before each use. All GFP-BAP biotinylation and L2-BirA infection experiments herein were performed with fresh aliquots of virus, at a non-saturating multiplicity of infection (MOI) according to the curve in Fig 1F.

To ensure that GFP-BAP biotinylation results only from encapsidated BirA protein and not from expression of trace amounts of the 9.5 kb L2-BirA plasmid that may have been packaged during PsV production, HaCaT GFP-BAP cells were infected with L2-BirA particles under conditions where nascent protein synthesis was blocked, either with actinomycin D to inhibit mRNA transcription or cycloheximide to block translation (S1A Fig). Inhibition of either transcription or translation did not alter the levels of GFP-BAP biotinylation (S1B and S1C Fig), but caused a drastic reduction in luciferase expression (S1C Fig). Thus, the translocation signal is due to L2-BirA protein from incoming capsids rather than nascent L2-BirA synthesis.

### L2 translocation precedes infection and requires endosome acidification and cyclophilin activity

**Fig 2:**
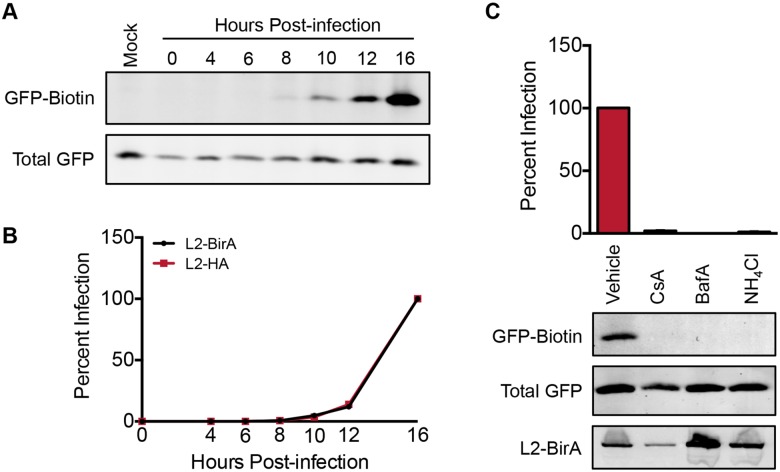
L2 translocation requires endosome acidification and cyclophilin activity. **(A)** Representative translocation blot from asynchronous HaCaT-GFP-BAP cells infected with 400 ng L1/ml of L2-BirA pseudovirions for indicated times. **(B)** Infection levels in HaCaT GFP-BAP cells infected with 300 ng L1/ml of L2-BirA or L2-HA PsV for the indicated times. Infection levels are expressed relative to the 16 hour time point, which is set at 100%. **(C)** Infection and translocation in HaCaT GFP-BAP cells infected with L2-BirA PsV in the presence of vehicle, the cyclophilin inhibitor cyclosporin A (CsA), or the endosomal acidification inhibitors bafilomycin A (BafA) and ammonium chloride (NH_4_Cl). Infection levels are expressed relative to vehicle-treated cells, which are set at 100%. All infection values represent mean percent infection (±SEM, *n* = 2), normalized to GAPDH.

To examine the kinetics of L2-BirA translocation and infection, we monitored biotinylation and luciferase expression in HaCaT GFP-BAP cells over time. GFP-BAP biotinylation was first detectable at 8 hours post-infection (Fig 2A). Luciferase expression in L2-BirA-infected cells was first observed at 10 hours post-infection and the infection profile was virtually indistinguishable from that of an L2-HA PsV (Fig 2B). Thus, translocation signal preceded reporter expression from the luciferase-expressing vDNA as expected, and infection kinetics of the virus are not affected by the large L2-BirA fusion. Prior work has shown that EdU-labeled vDNA signal partitions from L1 capsid somewhere between 6-12h post infection [23] and L2 localizes to the TGN somewhere between 8-16h post infection by proximity ligation assay [25]. Thus, the faint detection of L2 translocation at 8h in our system is consistent with the timing of L2/vDNA arrival at the TGN.

Uncoating is an important step in HPV entry that releases the L2/vDNA complex from the L1 capsid to allow for subsequent trafficking to the TGN and the nucleus [43]. Breakdown of the HPV capsid requires endosome acidification [15, 19, 20] and dissociation of L2/vDNA from L1 pentamers requires the activity of host cyclophilins [22]. As a verification of the L2-BirA assay, we examined how L2 translocation is affected by conditions that block uncoating and L1/L2 dissociation. Inhibitors of both endosome acidification and cyclophilins strongly blocked luciferase expression and GFP-BAP biotinylation (Fig 2C). Internalization of L2-BirA virus, as measured by L2 immunoblotting of alkaline-washed infected cell lysates, was unaffected by these treatments. These data substantiate the validity of the L2-BirA assay, since L2 translocation should require prior breakdown of the capsid structure.

### L2 translocation requires trafficking to the TGN in a furin- and γ-secretase-dependent manner

**Fig 3:**
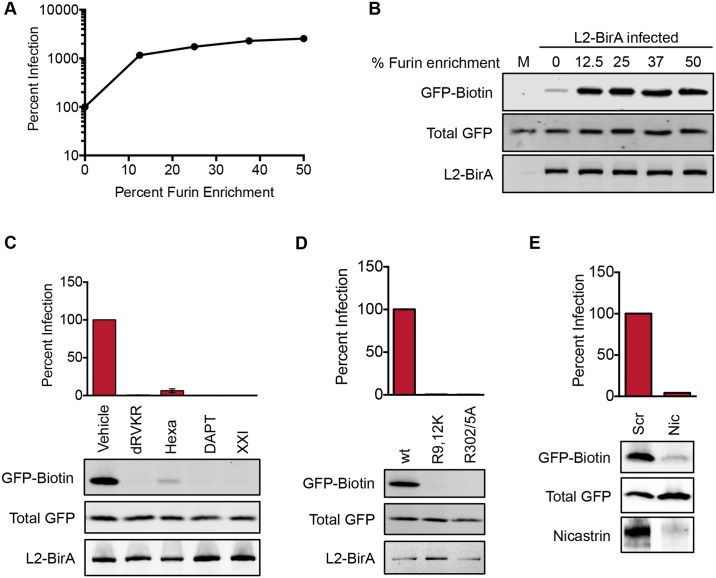
L2 translocation requires furin and γ-secretase activity. **(A)** Infection and **(B)** translocation of HaCaT GFP-BAP cells infected with L2-BirA PsV in the presence of varying amounts of furin enriched media. Infection values are expressed relative to 0% furin enrichment, which is set at 100% infection. **(C)** Infection and translocation in HaCaT GFP-BAP cells infected with L2-BirA PsV in the presence of vehicle, furin inhibitors dRVKR and hexa-D-arginine (Hexa) or γ-secretase inhibitors DAPT and XXI. Infection values are expressed relative to vehicle-treated cells, which are set at 100%. **(D)** Infection and translocation in HaCaT GFP-BAP cells infected with wt or mutant L2-BirA PsV. Infection values are expressed relative to wt L2-BirA infected cells, which are set at 100% **(E)** Percent infection and translocation in HaCaT GFP-BAP cells infected with L2-BirA PsV 40 hours post-treatment with scramble or nicastrin siRNAs. Infection values are expressed relative to scramble-treated cells, which are set at 100%. All infection values represent mean percent infection (±SEM, *n* = 2–3), normalized to GAPDH.

Cleavage of L2 by the cellular protease furin is critical for HPV infection. Furin cleavage of L2 occurs primarily on the cell surface, but is not required for virus binding or uptake into the endosomal compartment [42, 44]. L2 cleavage is essential for proper trafficking of L2/vDNA to the TGN, and based on this observation membrane penetration of L2 into the cytosol has been proposed to occur post-TGN localization [23]. We therefore tested the role of furin cleavage in L2 translocation. Addition of exogenous furin in the culture media resulted in a dose-dependent increase in both infection (Fig 3A) and GFP-BAP biotinylation, without a concomitant increase in virus uptake (Fig 3B) Conversely, GFP-BAP biotinylation was completely blocked in the presence of biochemical furin inhibitors (Fig 3C) or upon mutation (R9,12K) of the consensus furin cleavage site in L2 (Fig 3D).

Trafficking of L2/vDNA to the TGN also requires the multi-protein γ-secretase complex. Pharmacological inhibition of γ-secretase activity blocks HPV infection by preventing L2/vDNA from reaching the TGN [25]. We observed a potent inhibition of GFP-BAP biotinylation in cells that were infected in the presence of two different γ-secretase inhibitors (Fig 3C). Similar results were seen in samples where expression of the γ-secretase component nicastrin was knocked down for 40 hours prior to infection with L2-BirA (Fig 3E). Cumulatively, these results indicate that furin cleavage of L2 and γ-secretase activity are necessary for L2 translocation. Given the importance of furin and γ-secretase for trafficking of L2/vDNA to the TGN, these data indicate that L2 translocation occurs post-TGN localization. Further, the previously characterized L2 R302/305A mutation that causes retention of L2/vDNA within the TGN [37] also inhibited GFP-BAP biotinylation (Fig 3D), suggesting that proper egress from the Golgi is necessary for L2 translocation to occur. Notably, in the accompanying manuscript, the R302/5A mutation has also been shown to potently block association of L2-GFP fusions with mitotic chromatin in nocodazole-arrested cells [34], suggesting a potential connection between L2 chromosomal binding, TGN egress, and translocation.

During the course of this work, we observed some interesting anomalies with siRNA transfection in the BirA translocation system. Initial siRNA experiments utilized a transfection protocol that our lab had previously developed for use in HaCaT cells [45]. In this protocol, when HaCaT GFP-BAP cells were infected with L2-BirA virus at 24 hours post-transfection, nicastrin siRNAs potently blocked infection with the L2-BirA virus (S2A Fig). However, no corresponding decrease in GFP-BAP biotinylation was detected (S2B Fig). These seemingly contradictive results led us to hypothesize that a temporary perturbation of membrane integrity created by cationic lipid-based transfection reagents [46, 47] may cause aberrant translocation signal by leakage of GFP-BAP into vesicular compartments, which would for allow non-physiological interactions between L2-BirA and GFP-BAP. Indeed, GFP-BAP biotinylation still occurred in siRNA-transfected cells that were infected with L2-BirA in the presence of γ-secretase inhibitor XXI (S2C Fig), which causes a potent block in L2 translocation in the absence of siRNA transfection (Fig 3C). GFP-BAP biotinylation was also observed in siRNA-transfected cells that were infected with L2-BirA virus containing the R302/305A Golgi-retention mutant, which is normally unable to translocate (S2D Fig). Combined, these findings demonstrate that non-physiological L2 translocation signal can be induced during transfection. These issues were overcome by increasing the length of time between transfection and infection from 24 to 40 hours and adding additional washes to ensure removal of the transfection reagent (compare S2A and S2B Fig with Fig 3E). These experiments illustrate that care must be used when combining the L2-BirA assay with reagents that potentially perturb lipid membrane integrity and suggest that targets with short-lived knock down effects are not suitable for use with the system. However, these findings also demonstrate the sensitivity of the L2-BirA assay as a measure of L2-BirA and GFP-BAP compartmentalization.

### Cell cycle arrest blocks L2 translocation and causes vDNA to accumulate at the TGN

**Fig 4:**
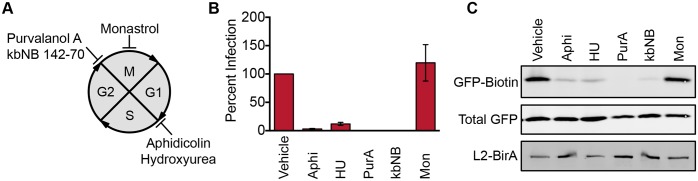
Cell cycle arrest blocks L2 translocation. **(A)** Diagram depicting where the inhibitors used in this study block cell cycle progression: aphidicolin (Aphi), hydroxyurea (HU), purvalanol A (PurA), kbNB 142–70 (kbNB), or monastrol (Mon). **(B)** Percent infection and **(C)** translocation in HaCaT GFP-BAP cells infected with L2-BirA in the presence of various cell cycle inhibitors. Infection values represent mean percent infection (±SEM, *n* = 3), normalized to GAPDH and expressed relative to vehicle-treated cells, which are set at 100%.

Prior research has demonstrated that inhibitors of the cell cycle abrogate PsV infection and block nuclear localization of the L2/genome complex [31–33]. We tested different cell cycle inhibitors in the L2-BirA assay, with the expectation that L2 translocation would still occur, since movement into the nucleus is presumed to be a post-translocation event. Aphidicolin and hydroxyurea were used to arrest cells in S phase; purvalanol A and kbNB 142–70 were utilized to block cell cycle progression at late G2; and monastrol, was used to arrest cells in mitosis (Fig 4A). Cell cycle status was analyzed by flow cytometry to ensure that all of the inhibitors behaved as expected (S3 Fig). As previously reported [31, 32], all of the inhibitors except monastrol blocked HPV infection (Fig 4B). Surprisingly, the S and late G2 inhibitors also potently inhibited GFP-GAP biotinylation (Fig 4C). Only monastrol, which arrests cells in mitosis after nuclear envelope breakdown, permitted biotinylation to occur at levels similar to those seen in cells treated with the vehicle DMSO.

**Fig 5:**
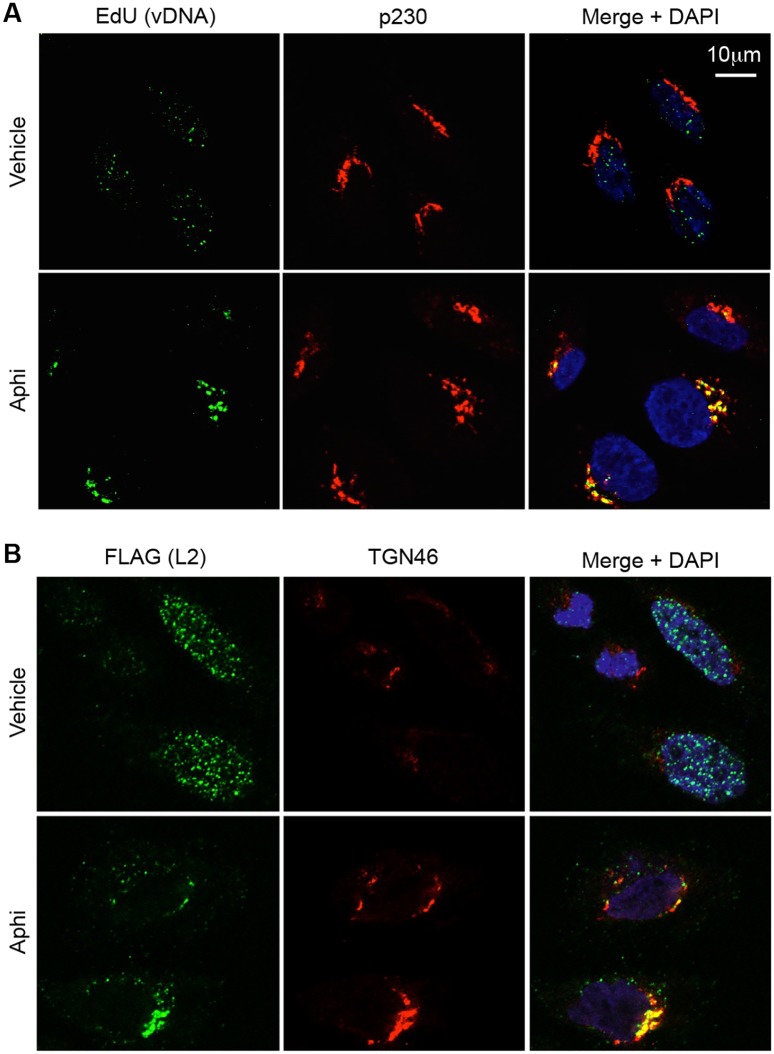
Cell cycle arrest traps vDNA and L2 in the TGN. **(A)** Representative image slices (0.35 μm thick) of HaCaT cells infected with wt PsV containing EdU-labeled DNA. Scale bar represents 10 μm. After fixation the cells were stained with Alexa Fluor 488 azide to visualize vDNA (green), p230 for the TGN (red) and DAPI to visualize nuclei (blue). **(B)** Representative image slices (0.35 μm thick) of HaCaT cells infected with wt L2-3xFTHA PsV and stained for FLAG (green), the TGN marker TGN46 (red) and DAPI to visualize nuclei (blue).

The translocation block observed above suggests that cell cycle inhibitors may impede or alter normal intracellular trafficking of L2 and prevent it from gaining access to the cytosol. Since L2 traffics in complex with the viral genome, we examined where vDNA localized in cell cycle arrested cells. HaCaT cells were infected with wt HPV16 containing EdU-labeled vDNA to allow for direct detection of vDNA by confocal microscopy. In vehicle-treated cells vDNA was readily detectable in both the TGN and nuclei of infected cells (Fig 5A). However, when cells were infected in the presence of the S phase inhibitor aphidicolin, vDNA was almost undetectable in the nucleus. Instead, consistent with prior observations [31], S phase arrest caused the vDNA to accumulate with the TGN protein p230. A similar accumulation of L2 with the TGN protein TGN46 was observed in aphidicolin-treated cells infected with PsV containing epitope-tagged L2-3xFLAG-thrombin-HA (L2-3xFTHA) (Fig 5B). TGN46 was used for experiments with L2-3xFTHA virus due to species incompatibility between the FLAG and p230 antibodies, which were both derived from mice. Collectively, the western blot and microscopy data suggest that inhibiting cell cycle progression blocks translocation of L2/vDNA, causing accumulation at the TGN.

### L2-BirA adopts a membrane-spanning topology during infection

L2 contains several short peptide motifs that mediate interactions with various cytosolic sorting factors to facilitate proper subcellular and retrograde trafficking of L2/vDNA from endosomes to the TGN [28, 29]. We have previously identified a critical transmembrane domain (TMD) upstream of the sorting motifs near the N-terminus of L2 (S4A Fig) [48], suggesting that L2 spans cellular membranes to expose these sorting motifs to their cytosolic interactions partners. A recent report used L2 immunofluorescence microscopy with selective plasma membrane permeabilization to confirm that L2 can indeed use this TMD during infection to span vesicular membranes with a lumenal N-terminal domain (residues 1–45) and residues immediately downstream of the TMD (residues 68–170) residing in the cytosol, which is consistent with, but not proof of, L2 adopting a type-I transmembrane protein topology [49]. This report also performed trypsin digestion assays with the L2 Golgi retention mutant of HPV18 (R295/298A, which is equivalent to the R302/305A mutant for HPV16 L2) to show that L2 spans across the TGN membrane, and that endosome acidification is required for L2 to span host membranes [49]. However, the previous study did not thoroughly determine the topology of the extreme C-terminus of L2, leaving open the possibility that C-terminal portions of L2 could be lumenal at the TGN. Indeed, S-phase block traps the L2/vDNA complex at the TGN but also potently blocks GFP-BAP biotinylation (Figs 4 and 5), suggesting that the BirA portion of the L2-BirA fusion is not exposed to the cytosol under these conditions. Moreover, if the C-terminal BirA fusion were exposed to the cytosol during early trafficking, we would expect to see translocation signal appear en route to the TGN, much earlier than 8 hours (Fig 2A).

One potential explanation is that the BirA fusion totally prevents L2 from spanning across host membranes. To test this hypothesis, we performed a trypsin digestion assay on HaCaT GFP-BAP cells infected with L2-BirA in the presence of DMSO, aphidicolin, or ammonium chloride (experimental details outlined in S4B Fig). At 22 hours post-infection external virus was washed off and the plasma membrane disrupted by passage through a needle under hypotonic conditions. Lysates were then treated with trypsin alone (to degrade cytosolically exposed proteins) or trypsin plus Triton X-100 (TX-100, to degrade cytosolic and lumenal proteins). When the samples were probed with BirA-specific antibody, L2-BirA was nearly undetectable in the trypsin-only samples treated with vehicle or aphidicolin (S4C and S4D Fig), suggesting that L2 has been degraded because it is in a membrane-spanning conformation with the bulk of the protein exposed to the cytosol. In contrast, the levels of L2-BirA detected in ammonium chloride treated cells were significantly higher than those seen with vehicle or aphidicolin, indicating that ammonium chloride treatment largely prevented L2-BirA degradation in the trypsin-only sample. Staining for the ER lumenal protein BiP showed significant degradation only in samples containing TX-100 plus trypsin (S4C Fig), indicating that vesicular membrane integrity was largely maintained throughout the assay. In summary, these data confirm the findings of DiGuiseppe et al. [49] and indicate that, similar to wt L2, L2-BirA adopts a membrane-spanning conformation post-endosome acidification. The fact that L2-BirA fails to give translocation signal pre-Golgi or in the presence of cell cycle inhibitors is indirect evidence that both the N- and C-termini of L2-BirA (including the BirA moiety) are indeed lumenal in this membrane-spanning conformation, a notion that will require further experimentation to confirm.

### L2/genome egress from the TGN requires progression into mitosis

**Fig 6:**
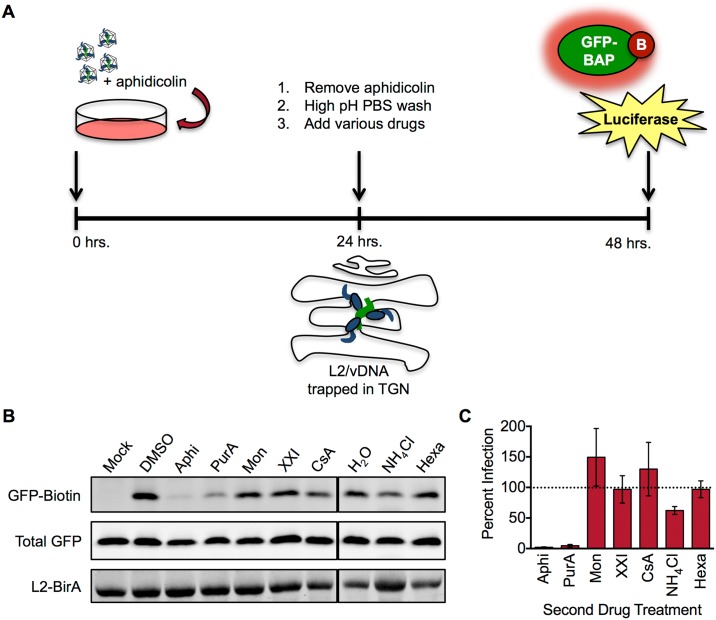
Mitotic progression is sufficient for L2 translocation to occur. **(A)** Schematic of the aphidicolin washout assay. **(B)** Representative translocation blot in the presence of various drugs post-aphidicolin washout. **(C)** Infection levels in the presence of various drugs post-aphidicolin washout. Values represent mean percent infection (±SEM, *n* = 3), normalized to GAPDH and expressed relative to vehicle-treated cells, which are set at 100%.

Several pieces of evidence suggest that cell cycle arrest prevents L2/vDNA trafficking to the nucleus by preventing the complex from trafficking past the TGN. We therefore designed an experiment to test whether cell cycle progression is sufficient for L2/vDNA to exit the TGN, penetrate the limiting membrane, and traffic to the nucleus (Fig 6A). Briefly, HaCaT GFP-BAP cells were infected in the presence of aphidicolin to allow L2/vDNA to accumulate at the TGN. At 24 hours post-infection, aphidicolin and external virus were washed off, and different drugs were added. The infection was allowed to proceed for an additional 24 hours before samples were collected to analyze translocation and infection. When cells were treated with drugs that inhibit HPV infection at points prior to Golgi localization, biotinylation levels were similar to those of vehicle-treated samples (Fig 6B), indicating that L2-BirA can now access GFP-BAP in the cytosol. Infection levels were also restored under these conditions (Fig 6C), indicating that the vDNA successfully egressed from the TGN to the nucleus. These results demonstrate that once the L2/vDNA complex reaches the Golgi, it has trafficked beyond its need for early infection requirements such as endosome acidification, cyclophilins, furin, and γ-secretase. However, GFP-BAP biotinylation and infection were still inhibited in cells that received a second aphidicolin treatment and thus remained in S phase arrest. GFP-BAP biotinylation and infection were also blocked by purvalanol A treatment, suggesting that progression from S to late G2 was not sufficient for L2 translocation to occur. In contrast, monastrol treatment restored GFP-BAP biotinylation and infection to levels seen in vehicle-treated samples. These findings indicate that cell cycle progression through the early stages of mitosis is sufficient for L2/vDNA to egress from the TGN.

### L2 translocation coincides with mitosis

**Fig 7:**
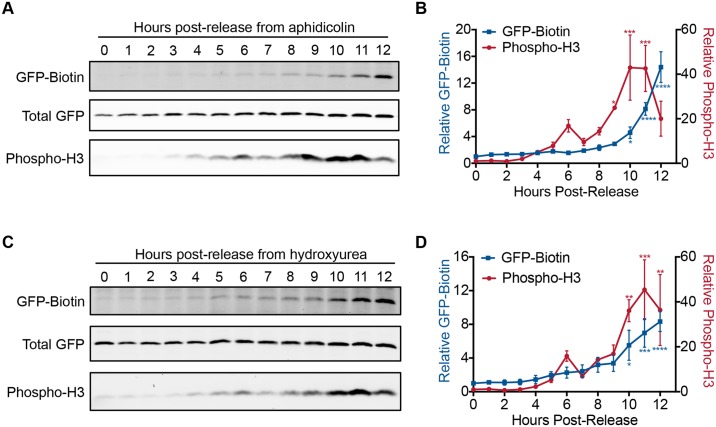
Time course of translocation in synchronized cells. HaCaT GFP-BAP cells infected with L2-BirA in the presence of aphidicolin or hydroxyurea to arrest cells at the G1/S transition. At 12 hours post-infection, the inhibitors were washed out and the cells were processed for SDS-PAGE at the indicated time points. **(A, C)** Representative translocation and phospho-H3 western blots. **(B, D)** Densitometry values represent mean GFP-biotin or phospho-H3, normalized to total GFP and expressed relative to the 0 hour time point (±SEM, *n* = 3). *P<0.05, **P<0.01, ***P<0.001, ****P<0.0001 as compared to the 0 hour time point. Blue asterisks denote significant differences in GFP-biotin levels; red asterisks denote significant differences in phospho-H3 levels.

Since progression into mitosis appears sufficient for L2 to breach the limiting membrane, we hypothesized that translocation would coincide with entry into mitosis. We therefore performed cell cycle synchronizations to examine when L2 translocation occurred relative to mitosis. To do this, we infected HaCaT-GFP-BAP cells with L2-BirA virus in the presence of aphidicolin to halt cells in S phase. At 12 hours post-infection, the aphidicolin was washed out, and the cell cycle was allowed to continue. Mitotic progression was monitored by phosphorylation of histone H3 at serine 10, a modification that is necessary for initiating chromatin condensation at the G2/M transition [50, 51]. After a small spike in phospho-H3 levels at 6 hours, phospho-H3 levels are significantly higher at 9 hours post-release, eventually tapering off around 12 hours post-release as the majority of the cell population exits mitosis (Fig 7A and 7B). GFP-BAP biotinylation levels correlate very well to phospho-H3 levels, gradually rising after release from aphidicolin, and are significantly higher than baseline at 10 hours post-release. Cells that were synchronized with a different S phase inhibitor, hydroxyurea, produced similar results (Fig 7C and 7D). Thus the timing of L2-BirA cytosolic exposure coincides with or slightly lags behind the onset of mitosis.

### Chemical dispersal of the Golgi is insufficient for L2 translocation

Fragmentation and dispersal of the Golgi apparatus is a necessary step in early mitosis [52, 53]. Given that cell cycle inhibition blocks L2 translocation and vDNA accumulation in the Golgi and that L2 translocation coincides with mitosis, we hypothesized that Golgi dispersal may be the trigger that causes L2/vDNA translocation across the limiting membrane. To test this hypothesis, cells were infected with L2-BirA in the presence of aphidicolin to inhibit cell cycle progression and cause retention of L2/vDNA at the Golgi. At 24h post-infection, the cells were switched to media containing aphidicolin plus the Golgi dispersing/fragmenting drugs (GDDs) golgicide A, brefeldin A, or nocodazole, and translocation was assessed by western blot. Treatment with GDDs did not cause an increase in translocation when compared to cells treated with the vehicle DMSO (S5A Fig). Imaging of aphidicolin/GDD-treated cells demonstrated that the GDDs are active in the presence of aphidicolin (S5B Fig) and that EdU-labeled vDNA from wt PsV is still localized at nocodazole-fragmented TGN vesicles (S5C Fig). Unfortunately the highly dispersed EdU signal from GCA- and BFA-treated cells was too faint for imaging. Thus, chemical induction of Golgi dispersal is not alone sufficient to induce L2/vDNA translocation.

### vDNA rapidly moves from the TGN to cellular chromosomes during mitosis

**Fig 8:**
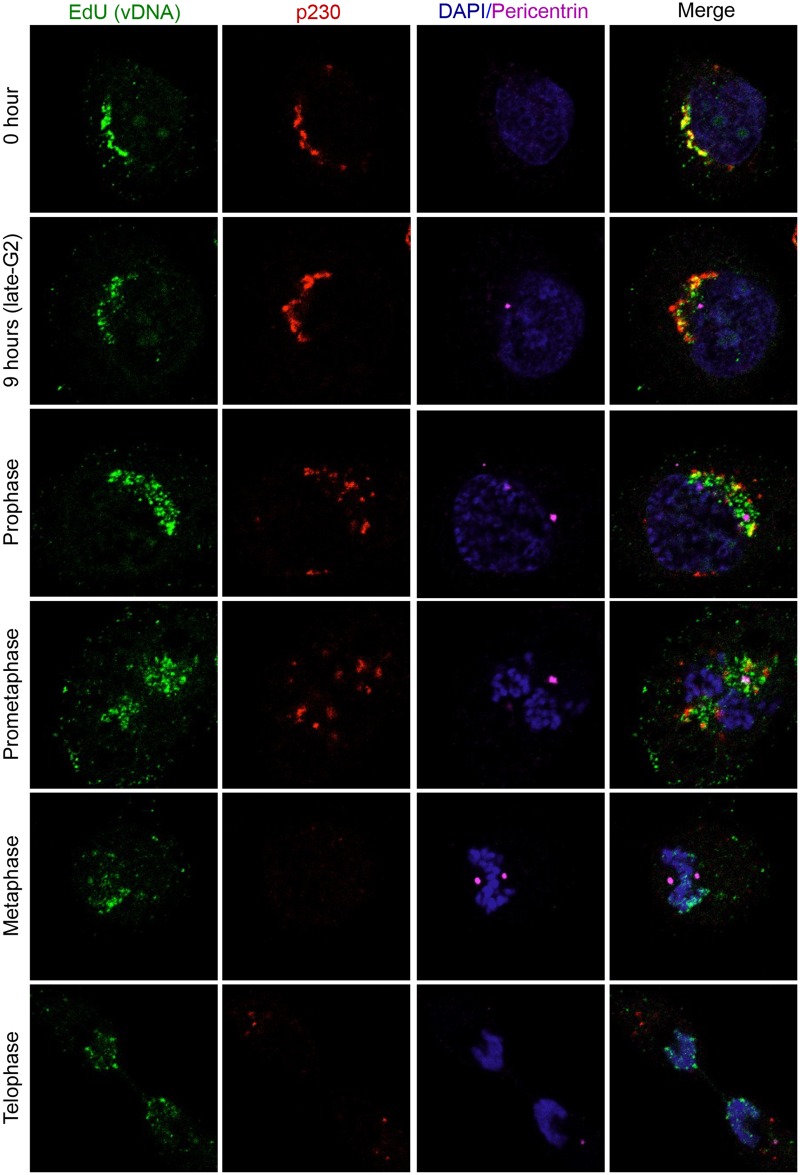
vDNA relocalizes from the TGN to condensed chromosomes during mitosis. HaCaT cells were infected with wt PsV containing EdU-labeled DNA in the presence of aphidicolin. At 24 hours post-infection, the aphidicolin was washed out and cells were fixed immediately or at 9 hours post-release. Cells were stained with AlexaFluor-488 azide (green) to visualize vDNA, p230 (red) to visualize the TGN, the centriole component pericentrin (magenta), and DAPI (blue). Individual channels and the merged images are representative image slices (0.35 μm).

Aydin et al. previously demonstrated that vDNA is detected on mitotic chromosomes from prometaphase to telophase with increasing association from prophase to metaphase, whereafter the association remained constant [31]. Thus, the localization of vDNA to host chromosomes is likely triggered by mitosis. We therefore followed the subcellular localization of EdU-labeled vDNA in cells released from aphidicolin synchronization. In interphase cells, EdU signal overlaps with p230, indicating that the viral genome is still localized at the TGN (Figs 8, S6 and S7). However, as the cells progress to prophase, there is a dramatic shift in EdU localization. As cells reach late G2, around 9 hours post release, the centrioles have begun to separate and EdU signal can start to be seen distinct from p230 positive structures. By prophase most of the costaining with the TGN is lost and instead most of the vDNA is now localized in the vicinity of the centrioles (Figs 8 and S6). After pericentriolar localization, the association of vDNA with mitotic chromosomes begins in prometaphase, and by metaphase the vast majority of vDNA is found decorating condensed mitotic chromosomes (Figs 8 and S7). The vDNA remains associated with host chromosomes throughout the remaining stages of mitosis. The microscopy data indicate that during mitosis the viral genome undergoes a dramatic change in subcellular localization, first moving from the TGN to a pericentriolar region, followed by another shift onto host chromosomes. Thus, similar to L2 translocation, vDNA movement from the TGN to host chromosomes coincides with mitosis.

## Discussion

A major barrier that all nonenveloped viruses must overcome is transport of their nucleic acid genomes across limiting membranes during infection of host cells. The papillomaviruses have evolved the L2 minor capsid protein, which complexes with the vDNA to ensure proper subcellular trafficking and subsequent penetration of limiting membranes. In this work we describe the development of the first direct assay for membrane translocation of L2, based on the bacterial biotin ligase BirA and its cognate BAP substrate. Our system involves the infection of GFP-BAP-expressing cells with virions encapsidating an L2-BirA fusion. Limiting membranes separate the lumenal virion-associated BirA from the cytosolic GFP-BAP substrate. Thus, biotinylation of GFP-BAP during infection is a direct consequence of L2 translocation across the limiting membrane. The enzymatic signal-amplifying nature of the system was intentional to favor the sensitive detection L2-BirA translocation. Similar BirA/BAP-based systems could be useful for trafficking and penetration studies of other viruses containing capsid proteins permissive to heterologous fusions, like adenovirus and the pIX capsid protein [54]. Likewise, we anticipate and are working towards extending the system to enable tracking of subcellular L2-BirA trafficking by engineering host cells to express BAP-tagged substrates in specific subcellular compartments.

L2-BirA PsV recapitulated the trafficking and infection kinetics of unmodified wt PsV and L2-BirA translocation was sensitive to pharmacological inhibitors that interfere with normal subcellular transport of L2/vDNA to the TGN (Figs 2 and 3). These data strongly suggest that localization to the TGN is a prerequisite to translocation, which has long been assumed, but never directly tested. Cell cycle arrest experiments revealed a strict requirement for progression into mitosis for L2-BirA translocation to occur (Fig 4). In agreement with previously published data [31], S phase block resulted in accumulation of vDNA at the TGN (Fig 5). Only upon release from S phase and entry into mitosis did vDNA egress from the TGN (Fig 8). Likewise, release from S phase was necessary to restore L2-BirA translocation (Fig 6). Synchronization timecourse experiments with two different S phase blockers, aphidicolin or hydroxyurea, revealed that translocation signal coincided with or lagged slightly behind the appearance of phosphorylated histone H3, a marker of progression into mitosis (Fig 7). Collectively these data strongly suggest that translocation occurs post-TGN localization and requires the onset of mitosis.

Given that the BirA translocation system is dependent on membrane compartmentalization, the topology of L2 in the TGN membrane has important implications for the interpretation of the assay. We previously identified a transmembrane domain (TMD) near the N-terminus of L2 that is essential for infection [48]. A recent report suggested L2 can span the TGN membrane in a topology consistent with a type I transmembrane protein, with residues immediately N-terminal of the TMD in the TGN lumen while residues immediately C-terminal of the TMD are cytosolic [49]. However, the topology of the extreme C-terminus of L2 was not directly assessed in this study or any others, leaving open the possibility that C-terminal portions of L2 remain lumenal at the TGN. This possibility has important implications for the L2-BirA system. If L2-BirA spans the TGN membrane in a type I topology as has been suggested, BirA should biotinylate cytosolic GFP-BAP prior to complete translocation. However, aphidicolin treatment, which traps L2/vDNA at the TGN (Fig 5), potently blocks GFP-BAP biotinylation (Fig 4C), indicating that L2-BirA does not have access to GFP-BAP at the TGN. Importantly, L2-BirA is still sensitive to trypsin digestion in the presence of aphidicolin (S4 Fig), demonstrating the BirA fusion does not prevent L2 from inserting into membranes. Combined, these data suggest that if L2-BirA assumes a type I topology at the TGN then the BirA portion may not be able to access its substrate, possibly due to steric hindrance from proximal association to the membrane or binding of cellular sorting factors to the C-terminal region of L2 [28, 29]. Alternatively, L2-BirA may span the TGN membrane twice, with both the N- and C-termini remaining lumenal. Future work is necessary to elucidate the precise topology, or topologies, that L2 adopts in cellular membranes. Regardless of its topology in the membrane, our data support a model where BirA cannot access cytosolic GFP-BAP until L2-BirA has passed the TGN and mitosis has commenced.

The Golgi apparatus undergoes dramatic and rapid changes during the transition from late G2 to early M phase. The initial fragmentation is an important checkpoint for progression through G2/M, and the eventual dispersal of Golgi into small cytosolic vesicles is believed to enable equal partitioning of Golgi elements between daughter cells [52, 55–57]. We initially hypothesized that these dynamic changes in Golgi morphology might be a trigger for L2 translocation. Contrary to our expectations, chemical fragmentation and dispersal of the Golgi was alone insufficient to cause L2-BirA translocation (S5 Fig). However, it cannot be ruled out that Golgi fragmentation in combination with processes unique to *bona fide* mitosis may be required for translocation to occur. Indeed, many of the factors required for mitotic Golgi dispersal are only activated in late G2/M [57, 58].

Direct observation of EdU-labeled vDNA during wt PsV infection of synchronized HaCaT cells corroborated our findings with the L2-BirA translocation system. These experiments indicated that vDNA traffics to host chromosomes in a biphasic manner. vDNA was initially colocalized with the TGN marker p230 during the aphidicolin block and at post-release times prior to mitosis (Figs 8, S6 and S7). During the transition from late G2 to early prophase the vDNA underwent a striking relocalization from the TGN to the pericentriolar region. These changes were concomitant with Golgi fragmentation, chromosome condensation, and centriole segregation (Fig 8). Given that centrosomes nucleate microtubule spindle formation during mitosis, it is reasonable to hypothesize that L2/vDNA traffics along portions of these microtubules. Indeed, some colocalization of vDNA with alpha tubulin-containing spindle-like structures was recently observed in PsV-infected mitotic cells [35]. In contrast, the accompanying manuscript by Aydin et al. observed efficient localization of transiently expressed L2-GFP fusion to mitotic chromosomes in the absence of microtubules [34]. Thus the question of whether L2/vDNA localization to host chromosomes requires microtubule transport remains unresolved, but the L2-chromosome interaction itself does not appear to require microtubules.

The transient pericentriolar distribution of vDNA lasted through prophase and into prometaphase, at which time the vDNA had begun a second relocalization to mitotic chromosomes (Figs 8 and S6). The vDNA was bound to chromosomes by metaphase, where it stayed throughout the rest of mitosis, partitioning into the daughter cells during anaphase/telophase (Figs 8 and S7). The distribution of vDNA on the condensed chromosomes was evocative of a physical interaction or tethering, supporting the hypothesis of earlier work by Aydin et al. [31]. Collectively the translocation and microscopy data support a model where L2/vDNA accumulate at the TGN of interphase cells and undergo a rapid relocalization to the centriolar region at the onset of mitosis in late G2/early prophase, followed by a shift to the condensed chromosomes by metaphase. However, several important questions remain: Does L2/vDNA traffic directly from the TGN to mitotic chromosomes, or does the complex pass through other intracellular compartments on its way to the nucleus? Furthermore, does visual egress from the TGN represent the translocation event? Or is the L2/vDNA complex still vesicle-bound during its pericentriolar localization?

Given the short duration of time between the onset of mitosis and pericentriolar localization of vDNA, it is reasonable to hypothesize that the viral genome takes a direct route from TGN to the nucleus. However, the possibility that L2/vDNA traffic to other sites before reaching the nucleus should not be ruled out. Day et al. observed slight overlap of HPV16 vDNA with more distal *cis/medial*-Golgi markers giantin and GM130 [23]. Likewise Ishii et al. reported partial colocalization of the related high risk HPV type 51 vDNA with the *cis*-Golgi marker GM130 [26]. L2 has also been suggested to retrograde traffic beyond the Golgi, to the ER based on proximity ligation assay with the lumenal ER residents BiP and protein disulfide isomerase [25]. Whether these represent primary or alternative routes of infection, or even unproductive dead ends is not clear.

During preparation of this manuscript, DiGiuseppe and colleagues published a paper hypothesizing that vDNA remains in a vesicular compartment during mitosis after egress from the TGN [35]. This hypothesis is based on nuclease protection assays and qPCR quantification of vDNA levels during infection, and dual fluor-azide conjugation of EdU-labeled vDNA in selectively and differentially permeabilized cells. They also present microscopy data with the L2 R302/305A Golgi retention mutant showing visual egress of this mutant from TGN markers during mitosis, but a failure to associate with mitotic chromosomes [35]. Their prior work with this mutant showed enhanced vDNA/TGN colocalization in interphase cells [37], suggesting this mutant is not really retained in the Golgi but may egress from the TGN during mitosis in a vesicle-bound state only to become reabsorbed into the reforming TGN of the daughter cells. Importantly, the companion paper by the Schelhaas group, using an assay based on transient L2-GFP expression, defines a novel chromatin binding region (CBR) in L2 (residues 188–334), that is necessary and sufficient to promote L2 association with mitotic chromosomes [34]. Mutagenesis experiments with this assay revealed that the R302/5A mutation as well as IVAL286AAAA and RTR313EEE substitutions caused a striking defect in mitotic chromosome binding. Like the R302/5A mutant, PsV containing the L2 RTR313EEE and IVAL286AAAA mutants reach the TGN, but fail to bind mitotic chromosomes [34]. In the accompanying manuscript these CBR mutations were tested for translocation in the L2-BirA system. Strikingly, the chromatin binding activity and the translocation ability of these CBR mutants are closely correlated; IVAL286AAAA, R302/5A, and RTR313EEE mutations cause complete abrogation of both chromatin binding and translocation (Fig 3D and [34]). These findings suggest that chromatin binding may be a necessary prerequisite for translocation of the L2/vDNA complex out of the TGN-derived vesicles. Additionally, the data presented here and in the companion paper demonstrate that the chromosomal association of L2-GFP [34] and incoming L2/vDNA complexes (Figs 8, S6 and S7) both begin specifically during prometaphase. Collectively, these data support the model of DiGiuseppe et al. that the egress of vDNA from the TGN precedes true translocation of the L2/vDNA complex and furthermore, that translocation is dependent on the L2 CBR binding an unknown mitotic chromosome tethering factor that becomes available specifically during prometaphase.

In summary we have developed a novel system to directly study translocation of the L2/vDNA complex during papillomavirus infection. We report, for the first time, that translocation requires TGN localization and entry into mitosis. Combined with microscopy of EdU-labeled vDNA and the work of others [34, 35], our findings support a model where L2/vDNA translocation occurs in mitosis after the visual egress of L2/vDNA from the TGN and is dependent on the chromatin binding ability of L2 via a newly defined CBR. Mitosis is an enormously dynamic and rapid process. Many drastic changes in subcellular structure and organization occur within a short time: Golgi fragmentation and dispersal, chromosome condensation, centriole segregation, spindle formation, nuclear envelope breakdown and remodeling of the ER [59]. These dramatic processes occur in a highly coordinated and interconnected fashion. Elucidating the specific mechanisms and host requirements for L2/vDNA translocation will be an exciting challenge given the dynamic complexity of mitosis.

## Materials and methods

### Biochemical inhibitors

See Table 1 for a list of biochemical inhibitors used in this study.

**Table 1:** Pharmacological inhibitors used in this study.

Compound	Solvent	Source (catalog #)	Working Concentration
Actinomycin D	DMSO	Sigma Aldrich (A9415)	0.5 μg/mL
Aphidicolin	DMSO	Santa Cruz Biotechnology (201535)	6 μM
Bafilomycin A	Methanol	Millipore (196000)	100 nM
Brefeldin A	DMSO	Sigma (B6542)	150 nM
Cycloheximide	DMSO	Sigma (C104450)	10 μg/mL
Cyclosporin A	DMSO	Millipore (239835)	10 μM
DAPT	DMSO	Tocris (2634)	10 μM
dRVKR	DMSO	Millipore (344930)	25 μM
Golgicide A	DMSO	Santa Cruz Biotechnology (215103)	7.5 μM
Hexa-d-Arg	Water	Millipore (344931)	50 μM
Hydroxyurea	PBS	Sigma (H8627)	2 mM
Inhibitor XXI	DMSO	Millipore (565790)	200 nM
KbNB 142–70	DMSO	Fisher (396210)	10μM
Monastrol	DMSO	Sigma (M8515)	50 μM
NH_4_Cl	Water	Santa Cruz Biotechnology (202936)	20 mM
Nocodazole	DMSO	Santa Cruz Biotechnology (3518)	5 μM
Purvalanol A	DMSO	Santa Cruz Biotechnology (224244)	12 μM

### Plasmid cloning

The pCIP-NES-GFP-BAP plasmid expresses cytosolic EGFP fused to the biotin acceptor peptide (BAP), a specific substrate for the biotin protein ligase BirA [41]. Briefly, The EGFP cDNA was PCR-amplified from pEGFP-N1 (Clontech) with primers encoding an N-terminally fused PKI-type NES (NSNELALKLAGLDI) [60] and a C-terminally fused BAP (GLNDIFEAQKIEWHE). The NES-GFP-BAP product was then NheI/BamHI cloned into the pCMV-IRES-Puro plasmid backbone, a derivative of the lentiviral expression plasmid pCIG [61], modified by insertion of a puromycin resistance gene downstream of the IRES sequence. pCMV-IRES-Puro was a kind gift of Felicia Goodrum, University of Arizona. pXULL-L2-mycBirA is a derivative of the HPV16 L1/L2 expression plasmid pXULL. Briefly, a codon optimized N-terminally myc epitope tagged BirA gene was PCR-amplified from pcDNA3.1-mycBioID [62] with primers introducing unique BspEI and SalI sites. This product was then cloned into pXULL-L2-BKS, an intermediate construct containing in frame BspEI, KpnI, and SalI sites for generating C-terminal fusions on L2. The R118G mutation of the mutant BirA was then repaired back to R118 by Quickchange XL-II mutagenesis (Agilent #200522) to regenerate wild type BirA. pcDNA3.1-mycBioID was a gift from Kyle Roux (Addgene plasmid # 35700). p16shell.L2-3xFLAG-thrombin-HA (L2-3xFTHA) was a kind gift of Dan DiMaio and is described in [25]. pXULL-L2-3xFTHA was constructed by PCR amplifying the 3xFTHA linker with BspEI and SalI sites introduced to clone into pXULL-L2-BKS as a C-terminal fusion. An additional KpnI site was engineered into the linker for fusion of inserts downstream of 3xFTHA. pXULL-L2-3xFTHA-BirA was generated by PCR amplifying BirA, flanked with KpnI and SalI sites for ligation downstream of the 3xFTHA linker within pXULL-L2-3xFTHA. pXULL-L2-HA was generated by introducing an HA tag fused to the C-terminus of L2 by PCR, followed by subcloning the L2-HA back into pXULL with NotI and SalI.

### Tissue culture

All cell lines were maintained at 37°C with 5% CO_2_ and passaged every 2–3 days. 293TT (a kind gift from Chris Buck, NCI, described in [63]) cells were cultured in Dulbecco’s modified Eagle medium (DMEM) with high glucose and supplemented with 10% bovine growth serum (BGS, HyClone SH30541.03) and antibiotic/antimycotic (Ab/Am, Sigma A5955). Furin-secreting CHO furΔ1 cells and furin deficient FD11 cells (kind gifts of Patricia Day and Steven Leppla respectively, both described in [64]) were maintained in high glucose DMEM with 10% fetal bovine serum (FBS, HyClone SH30396.03), supplemented with 200 μM L-proline and Ab/Am. Concentrated, active furin was isolated from CHOfurΔ1 conditioned media, diluted into FD11 conditioned media as previously described [42]. HaCaT cells (a kind gift from Anne Cress, University of Arizona, originally described in [65]) were grown in high glucose DMEM supplemented with 10% FBS and Ab/Am.

### Generation of NES-GFP-BAP HaCaT subclone

HaCaTs, plated to subconfluence in a 6-well plate were transfected with 2 μg pCIP-NES-GFP-BAP using Lipofectamine 2000 (Life Technologies). After 48 hours of culturing and expansion to a 10 cm dish, media was supplemented with 300–400 nM puromycin (HyClone) to begin selection of clones stably expressing cytosolically localized GFP-BAP. Cells were further cultured and expanded until most non-fluorescent clones died off. After one week of maintenance, surviving cells were pooled and sparsely plated in 10cm dishes to isolate clonal populations. Puromycin was gradually decreased from 400 nM to 200 nM during this selection. Individual clones were examined for GFP fluorescence and select clones were isolated using cloning rings. An ideal clone and was subsequently expanded and banked as HaCaT-GFP-BAP. These cells were maintained in DMEM supplemented with 10% FBS, 200 nM puromycin, and Ab/Am.

### Pseudovirus production

Mutant viruses were generated by PCR-based methods or by site-directed mutagenesis using the QuikChange XL-II kit. All mutations were verified by Sanger sequencing. PsV were generated as previously described [66]. Briefly, 293TT cells were CaCO_4_ co-transfected with the appropriate pXULL based plasmids and the luciferase reporter plasmid pGL3; virus was then purified by CsCl gradient. The L1/L2 content of purified PsV was verified by SDS-PAGE and Coomassie staining, as compared to bovine serum albumin protein standards. Pseudogenome content was determined by SYBR green qPCR using primers specific for the luciferase gene in pGL3. The capsid:genome ratios were all within the normal range for typical wild type HPV16 preps. L2-BirA PsV were generated from pXULL constructs encoding either L2-mycBirA or L2-3xFTHA-BirA fusions. These L2-BirA containing PsV had comparable performance in GFP-BAP translocation assays and were used interchangeably in this work. pXULL-L2-BirA constructs are 9.5 kb in size, too large to be efficiently packaged within the capsid using the 293TT PsV system [63, 67].

### *In vitro* BirA biotinylation

Purified recombinant maltose binding protein substrate (MBP-BAP) was obtained from Avidity (#BIS300). L2-BirA viruses were calculated to contain approximately 20 ng BirA per 500 ng L1, assuming 24 molecules L2-BirA per virion. To disassemble the L1 capsid and make L2-BirA accessible for reaction, 500 ng purified L2-BirA wt and R9,12K mutant PsV particles were reduced in VSB + 16.7 mM DTT for 4 hours at room temperature. 50 μL reactions were then set up in 50 mM bicine, 6.5 mM Tris, pH = 8.3, 10 mM ATP, 10 mM Mg-acetate, 50 μM D-biotin, 5 μg MBP-BAP substrate (unbiotinylated or biotinylated positive control, Avidity #BIS300), and 500 ng L1 (L2-BirA virus) of the DTT-reduced PsV. Reactions were stopped by the addition of SDS-PAGE loading buffer at 1, 4, and 16 hours. Samples were heated at 95°C for 6 minutes prior to SDS-PAGE and western blot for biotinylated MBP, total MBP, and L2-BirA.

### Infections

HaCaT GFP-BAP cells were plated at 50,000 cells per well in a 24-well plate. Cells were infected the following day with L2-BirA virus at 2 x 10^8^ viral genomes/well. At 24 hours post-infection (p.i.), the cells were washed once with PBS and lysed in 100 μl reporter lysis buffer (Promega E3971). Luciferase activity was measured on a DTX-800 multimode plate reader (Beckman Coulter) using luciferase assay reagent (Promega E4550) according to the manufacturer’s instructions. A fraction of these lysates were blotted for GAPDH to normalize luciferase activity.

### Translocation assays

Unless otherwise stated, HaCaT GFP-BAP cells were plated at 50,000 cells per well in a 24-well plate. The following day, the cells were infected with L2-BirA virus at 300 ng L1/ml. At 24 hours p.i., cells were lysed in 1X RIPA buffer (50 mM Tris-HCl pH 8.0, 150 mM NaCl, 1% NP40, 0.5% sodium deoxycholate, 0.1% SDS) supplemented with 1X reducing SDS-PAGE loading buffer, 1X protease inhibitor cocktail (Sigma P1860), 1mM PMSF and 1X PhosSTOP phosphatase inhibitor cocktail (Roche 04906845001). For experiments where intracellular levels of L2 were assayed, cells were washed prior to lysing with alkaline PBS, pH 10.7 for 2.5 minutes to strip non-internalized virus off of the cell surface [18]. The samples were boiled at 95°C for 5 minutes and stored at -80°C until further processing by western blot.

### Western blotting

Samples were resolved by SDS-PAGE and transferred onto a 0.45 μm nitrocellulose membrane for blotting. For GAPDH levels, blots were blocked in 5% non-fat powered milk dissolved in Tris-buffered saline containing 0.1% Tween (TBST) and stained with anti-GAPDH (Cell Signaling 2118) at 1:5000. To check for L2-BirA levels, blots were blocked in 5% milk/TBST and stained with anti-L2 K4 at 1:5000 or anti-BirA (abcam 14002) 1:2000. For translocation, blots were blocked in 100% Odyssey blocker buffer (Licor 927–40000) and stained sequentially with neutravidin DyLight 800 (Pierce 22853) and anti-GFP (Clontech 6323770) at 1:5000 in 50% Odyssey blocker buffer/TBST. The lower portion of some translocation blots was cut off and stained with anti-phopsho-H3 (Cell Signaling 3377) at 1:10,000 in 5% BSA/TBST. Nicastrin knockdown was verified by blotting with anti-nicastrin (Cell Signaling 5665) at 1:1000 in 5% bovine serum albumin (BSA)/TBST. MBP-BAP was stained using anti-MBP (New England Biolabs E8030) at 1:10,000 in 1% BSA/TBST. Goat anti-rabbit DyLight 680 (Pierce 35568), goat anti-mouse DyLight 680 (Pierce 35518), goat anti-mouse DyLight 800 (Pierce 35521), and goat anti-chicken DyLight 680 Pierce (10074) were used as secondary antibodies at 1:10,000 in 5% milk/TBST. Blots were imaged on the Licor Odyssey Infrared Imaging System. Band intensities were quantified by densitometry using ImageJ v1.48.

### siRNA experiments

Pooled scramble (sc-37007) and nicastrin (sc-36036) siRNA duplexes were obtained from Santa Cruz Biotechnologies. HaCaT GFP-BAP cells were plated in 24-well plates at 40,000 cells per well in Ab/Am-free DMEM/10% FBS. The next day, cells were washed once with PBS and the media replaced with OptiMEM (Life Technologies). Cells were transfected with 50 nM siRNA using Lipofectamine RNAiMax (Life Technologies 13778150) according to the manufacturer’s instructions. At 18 hours post-transfection, siRNA/transfection reagent were washed off by rinsing cells once with PBS and then adding Ab/Am-free DMEM/10% FBS to each well. Cells were infected at 24 hours or 40 hours post-transfection in Ab/Am-free DMEM/10% FBS. At 24 hours p.i., samples were collected for luciferase and western blotting as described above. For infections occurring at 40 hours post-transfection, cells were washed at two additional time points to further remove residual transfection reagent: 24 and 40 hours post-transfection.

### Cell cycle analysis

Cell cycle status was analyzed by propidium iodide (PI) incorporation and flow cytometry. Briefly, HaCaT GFP-BAP cells were treated with the appropriate inhibitors for 24 hours. Cells were collected by trypsinization and pelleted at 1000 rpm for 10 minutes at 4°C. The pellet was resuspended in ice cold 70% ethanol to fix the cells and stored at -20°C until ready for staining. For PI staining, cells were pelleted at 2000 rpm for 15 minutes at 4°C, resuspended in PBS, pH 7.4 containing 40 μg/mL PI and 500 μg/mL RNase A, and incubated at 37°C for 30 minutes. PI-stained cells were immediately analyzed using the BD Biosciences FACSCanto-II flow cytometer and Diva 8.0 software.

### EdU detection and immunofluorescence

Pseudovirions containing 5-ethynyl-2’-deoxyuridine (EdU) labeled pseudogenomes were produced by CaPO_4_ transfection of 293TTs supplemented with 15 μM EdU as previously described [45]. For infections, HaCaT cells were seeded on glass coverslips in 6-well plates at 100,000 cells per well. The following day, the cells were infected with 700ng of L1/ml of EdU-labeled virus in the presence of various drugs. At 16 hours p.i., cells were washed once with fresh media to remove unbound virus. Fresh media plus drugs were then added and the infection continued for an additional 14 hours. At 30 hour p.i., the cells were washed once in PBS, pH 7.4 (PBS), then 1 x 2.5 min in PBS, pH 10.7 to remove surface-bound virus, then washed twice in PBS, pH 7.4. In most cases the cells were fixed with 2% paraformaldehyde/PBS for 10 minutes at room temperature (RT) and permeabilized with 0.2% Triton X-100/PBS for 10 minutes at RT. In experiments where pericentrin was stained, the cells were fixed and permeabilized for 15 minutes in 100% methanol at -20°C. The samples were blocked in 4% BSA/1% goat serum/PBS overnight at 4°C. EdU-labeled vDNA was then conjugated to Alexa Fluor 488-N_3_ using click chemistry according to manufacturer’s instructions (Life Technologies C10337). For immunofluorescence, polyclonal rabbit anti-TGN46 (Sigma T7576), mouse anti-p230 (BD Biosciences 611280), mouse anti-GM130 (BD Biosciences 610822), mouse anti-FLAG (Sigma F3165) and rabbit anti-pericentrin (BioLegend 923701) were all used at 1:500. Alexa Fluor-488, -555 and -647 labeled goat anti-mouse and goat anti-rabbit secondary antibodies (Life Technologies A11029, A21424, A21429 and A21236) were used at 1:1,000. Coverslips were mounted on glass slides with Prolong Antifade Diamond containing 4′,6-diamidino-2-phenylindole (DAPI) (Life Technologies P36971).

### Confocal and epifluorescence microscopy

Confocal microscopy was performed using a Zeiss LSM510 META system with a 405 nm laser diode, a 488 nm argon laser and a 543 nm He/Ne1 laser or the Zeiss LSM880 system with 405 nm, 488 nm, 543 nm and 633 nm lasers. Samples were examined using a 63x objective, and Z-stacks with a 0.35μm depth per plane were taken of each image. Representative single-plane images were processed with the Zeiss META software or Zen Blue software and further processed with Microsoft PowerPoint software. Epifluorescent micrographs were taken using an Olympus IX71 inverted microscope with a 40x objective and xenon UV lamp.

### Transmission electron microscopy

Each sample was applied to an ultra-thin carbon film over Lacey Carbon Support Film on 400-mesh copper grids (Ted Pella, Inc.) that were glow discharged for 1 minute. Excess solution was blotted with Whatman #1 filter paper and the grid was rapidly stained with 2% uranyl acetate. The uranyl acetate was immediately blotted with Whatman #1 filter paper and rapidly stained with 2% methylamine tungstate. The second stain was immediately blotted away with Whatman #1 filter paper and allowed to air dry. Data was collected on a JEOL 3200FS microscope operated at 300 kV. Images were acquired at a magnification of 46,000x and at 1.0 μm underfocus using a Gatan UltraScan 4000 charge-coupled device (CCD) camera.

### Aphidicolin release into pharmacological inhibitors

HaCaT GFP-BAP cells were plated at 50,000 cells per well in a 24-well tissue culture plate. The following day, cells were infected on ice for 1 hour with wt L2-BirA virus at either 2 x 10^8^ viral genomes/well (for infection) or 150 ng L1/well (for translocation) in the presence of 6 μM aphidicolin. After one hour, unbound virus was washed off, fresh media containing 6 μM aphidicolin was added, and the cells were shifted to 37°C. After 24 hours, the aphidicolin was washed out by rinsing the cells one time with warm media, followed by one time with PBS, pH 10.7 for 2.5 minutes to remove non-internalized virus, and then one more time with warm media. Fresh media containing different biochemical inhibitors was then added to the appropriate wells and the infection was allowed to proceed for an additional 24 hours. At this point the samples were collected to measure infection by luciferase activity and translocation by GFP-BAP biotinylation, as described above.

### Infection with Golgi dispersing drugs

HaCaT GFP-BAP cells were plated at 50,000 cells per well. The following day, the cells were infected with 150 ng L1/well of wt L2-BirA virus in the presence of 6 μM aphidicolin. At 24 hours p.i., fresh media was added containing aphidicolin plus one of the Golgi dispersing drugs, or aphidicolin plus the vehicle DMSO. After 4 hours of GDD treatment, the samples were lysed and translocation assessed by western blotting as described above. To ensure that GDDs are still active in the presence of aphidicolin, a parallel set of cells on glass coverslips were similarly treated, processed for immunofluorescence as described above, and imaged by epipfluorescence microscopy. To test the effect of GDDs on vDNA trafficking, HaCaT cells were plated on coverslips in 6-well plates at 100,000 cells per well. The next day, the cells were infected with 700ng of L1/ml of EdU-labeled virus for a total of 30 hours in the presence of aphidocolin. At 30 hours p.i., fresh media containing aphidocolin plus nocodozole or the vehicle DMSO was added and the cells were cultured for an additional 4 hours. The samples were then processed for EdU detection and immunofluorescence as described above and imaged by confocal microscopy.

### Trypsin digestion assay

HaCaT GFP-BAP cells were plated at 150,000 cells per well in a 6-well plate. Cells were infected the following day with L2-BirA virus at 1 μg L1/well. At 22 hours p.i. the cells were washed once with PBS pH 7.2. The cells were then washed with PBS pH 10.75 for 2.5 minutes to remove surface bound virions. The cells were further washed 2x with PBS 7.2 to neutralize pH. Next, 0.05% trypsin-EDTA was added and cells were incubated for 15 minutes at 37°C to further remove surface bound virions and lift cells off the plate. The trypsin was neutralized with cDMEM and the cells were pelleted at 800 rpm for 5 minutes. Cells were then resuspended in 105 μl cold hypotonic lysis buffer (20 mM Tris-HCl, 10 mM EDTA, pH 8.5) and incubated on ice for 15 minutes. The cells were then passed 15 times through a 25 gauge x 5/8” needle to lyse the plasma membrane. Lysates were then split into four equal parts and exposed to final concentration of 0.04% trypsin-EDTA and 0.5% TX-100 or equivalent volumes of vehicle. The lysates were incubated at 37°C for 55 minutes. The lysates were then put on ice and a final concentration of 400 μg/ml trypsin inhibitor from soybean (Sigma T6522) was added to quench the trypsin. Reducing SDS-PAGE loading buffer supplemented with a final concentration of 1 mM PMSF and 1X protease inhibitor cocktail (Sigma #P1860) was added to the samples. Samples were then boiled for 6 minutes at 95°C and stored at -80°C until further processing by western blot.

### Cell synchronization and translocation time course

HaCaT GFP-BAP cells were plated at 45,000 cells per well in 24-well plates. The next day, fresh media containing 6 μM aphidicolin or 2 mM hydroxyurea was added to each well for 6 hours. Cells were then infected with wt L2-BirA virus at 150 ng L1/well in the presence of 6 μM aphidicolin or 2 mM hydroxyurea. At 12 hours p.i. (18 hours post-inhibitor addition), the synchronized cells were released by washing each well twice with phosphate buffered saline, pH 7.4 (PBS) and adding fresh media to each well. Samples were collected and processed by western blotting as described above.

### Statistics

Statistical analyses were performed using Prism 6 (GraphPad Software). Significant differences were determined by one-way ANOVA followed by Dunnett’s multiple comparisons test or two-way ANOVA followed by Tukey’s multiple comparisons test.

## Supporting information

S1 Fig GFP-BAP is biotinylated by L2-BirA from incoming capsids. **(A)** Schematic of the experimental setup. To minimize toxicity, an abbreviated 14 hour infection was performed, with addition of actinomycin D (ActD) or cycloheximide (CHX) at 8.5 hours after the start of infection, with the cells only exposed to drugs for 5.5 hours. **(B)** Representative translocation blots for HaCaT GFP-BAP cells infected with L2-BirA PsV in the presence of vehicle (DMSO), ActD, or CHX. **(C)** Infection and translocation levels in the presence of vehicle or inhibitors. Infection values represent mean percent infection (±SEM, *n* = 2), normalized to GAPDH. Percent translocation levels were quantified by densitometry of GFP-biotin bands, normalized to total GFP band intensity. Percent infection and translocation are expressed relative to DMSO-treated cells infected with L2-BirA, which are set at 100%.
(TIFF)

S2 Fig Transfection reagent causes aberrant translocation signal. **(A)** Infection and **(B)** translocation in HaCaT GFP-BAP cells that were transfected with scramble (scr) or nicastrin (nic) specific siRNA for 24 hours and then infected with wt L2-BirA for an additional 24 hours. Infection values represent mean percent infection (±SEM, *n* = 2), normalized to GAPDH and expressed relative to scramble-treated cells, which are set at 100%. **(C)** Translocation in HaCaT GFP-BAP cells treated with media, the transfection reagent RNAiMax alone, or RNAiMax-conjugated siRNAs in the presence of the vehicle DMSO or γ-secretase inhibitor XXI. **(D)** Infection and translocation in HaCaT GFP-BAP treated with media or scramble siRNA for 24 hours and then infected with wt L2-BirA or R302/5-BirA for an additional 24 hours. Infection values represent mean percent infection (±SEM, *n* = 2), normalized to GAPDH and expressed relative to the wt sample for each condition, which are set at 100%.
(TIFF)

S3 Fig Verification of the cell cycle inhibitors used in this study. Flow cytometry of HaCaT GFP-BAP cells treated with various cell cycle inhibitors or vehicle control for 24 hours, fixed and analyzed for DNA content by propidium iodide. G1, S, and G2/M peaks are indicated on the vehicle (DMSO) profile.
(TIFF)

S4 Fig L2-BirA adopts a transmembrane topology post-endosome acidification. **(A)** Diagram of L2-BirA fusion protein showing furin cleavage site and transmembrane domain (TMD). **(B)** Diagram of the trypsin digestion assay experimental setup. Briefly, HaCaT GFP-BAP cells were infected with L2-BirA PsV for 22 hours in the presence of DMSO, Aphi, or NH_4_Cl. Cells were then washed with alkaline PBS and trypsinized to remove extracellular virus and lift the cells from the dish. Cells were gently pelleted and lysed by shearing. Crude lysate was aliquoted equally among four tubes for treatment ± trypsin and TX-100, and then incubated for 55 minutes at 37°C prior to processing for SDS-PAGE and western blot. **(C)** Anti-BirA and anti-BiP stains of infected cell lysates, treated as indicated. **(D)** Densitometry values represent mean L2-BirA levels, normalized to total BiP and expressed relative to the -trypsin condition for vehicle, Aphi and NH_4_Cl (±SEM, n = 3).
(TIFF)

S5 Fig Chemical disruption of the Golgi is insufficient to induce translocation. **(A)** Representative translocation blot of HaCaT GFP-BAP cells infected in the presence of aphidicolin for 24 hours and then treated with aphidicolin plus GDDs for 4 additional hours. **(B)** Representative epifluorescent images of HaCaT cells treated with aphidicolin for 24 hours and then treated with aphidicolin plus GDDs for an additional 4 hours. Cells were stained with anti-GM130 (green, *cis*-Golgi marker) and TGN46 (red, *trans*-Golgi marker). **(C)** Representative slices (0.35 μm) of HaCaT cells infected with wt PsV containing EdU-labeled DNA in the presence of aphidicolin for 30 hours, then exposed to aphidicolin plus nocodozole for 4 hours. After fixation the cells were stained with Alexa Fluor 488 azide to visualize vDNA (green), anti-p230 for the TGN (red), and DAPI to visualize nuclei (blue).
(TIFF)

S6 Fig vDNA relocalizes from the TGN to a pericentriole region early in mitosis. Maximum intensity projections of HaCaT cells infected with wt PsV containing EdU-labeled DNA in the presence of aphidicolin and processed as described in Fig 8. Representative images of interphase cells (0 hours post-release) and late G2 and prophase (9 hours post-release) are shown. White arrows indicate location of pericentrin.
(TIFF)

S7 Fig vDNA relocalizes from centrioles to host chromosomes in late mitosis. Maximum intensity projections of HaCaT cells infected with wt PsV containing EdU-labeled DNA in the presence of aphidicolin and processed as described in Fig 8. Representative images of prometaphase and meta/telophase cells (9 hours post-release) are shown. White arrows indicate location of pericentrin.
(TIF)
