# Supplementary figures and images for: Translocation of the papillomavirus L2/vDNA complex across the limiting membrane requires the onset of mitosis
Source: PLoS Pathog. 2017 May 2;13(5):e1006200. doi: 10.1371/journal.ppat.1006200 (PMC5412990; doi:10.1371/journal.ppat.1006200)

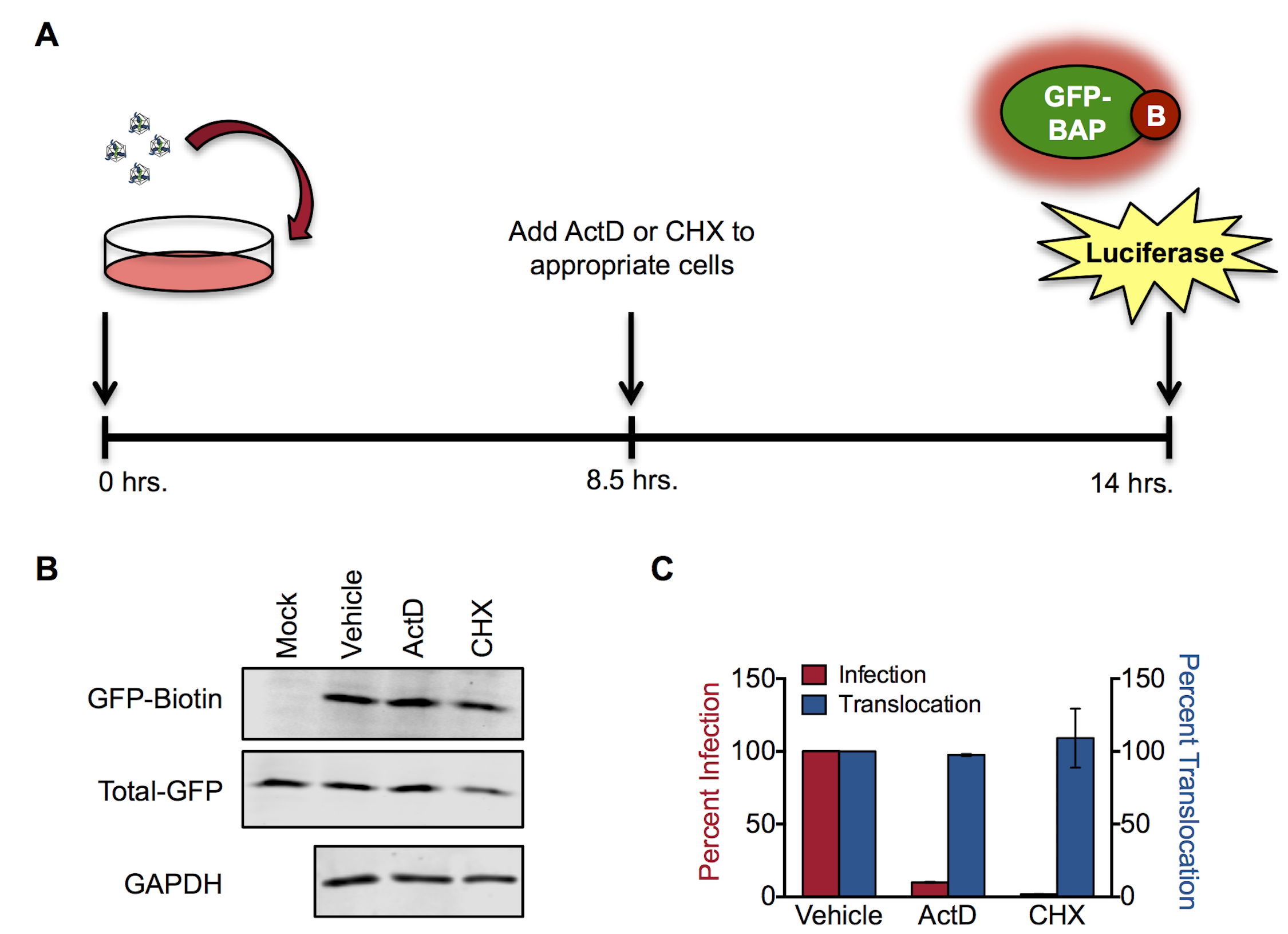

Supplement: S1 Fig — (A) Schematic of the experimental setup. To minimize toxicity, an abbreviated 14 hour infection was performed, with addition of actinomycin D (ActD) or cycloheximide (CHX) at 8.5 hours after the start of infection, with the cells only exposed to drugs for 5.5 hours. (B) Representative translocation blots for HaCaT GFP-BAP cells infected with L2-BirA PsV in the presence of vehicle (DMSO), ActD, or CHX. (C) Infection and translocation levels in the presence of vehicle or inhibitors. Infection values represent mean percent infection (±SEM, n = 2), normalized to GAPDH. Percent translocation levels were quantified by densitometry of GFP-biotin bands, normalized to total GFP band intensity. Percent infection and translocation are expressed relative to DMSO-treated cells infected with L2-BirA, which are set at 100%. (TIFF) [file ppat.1006200.s001.tiff]

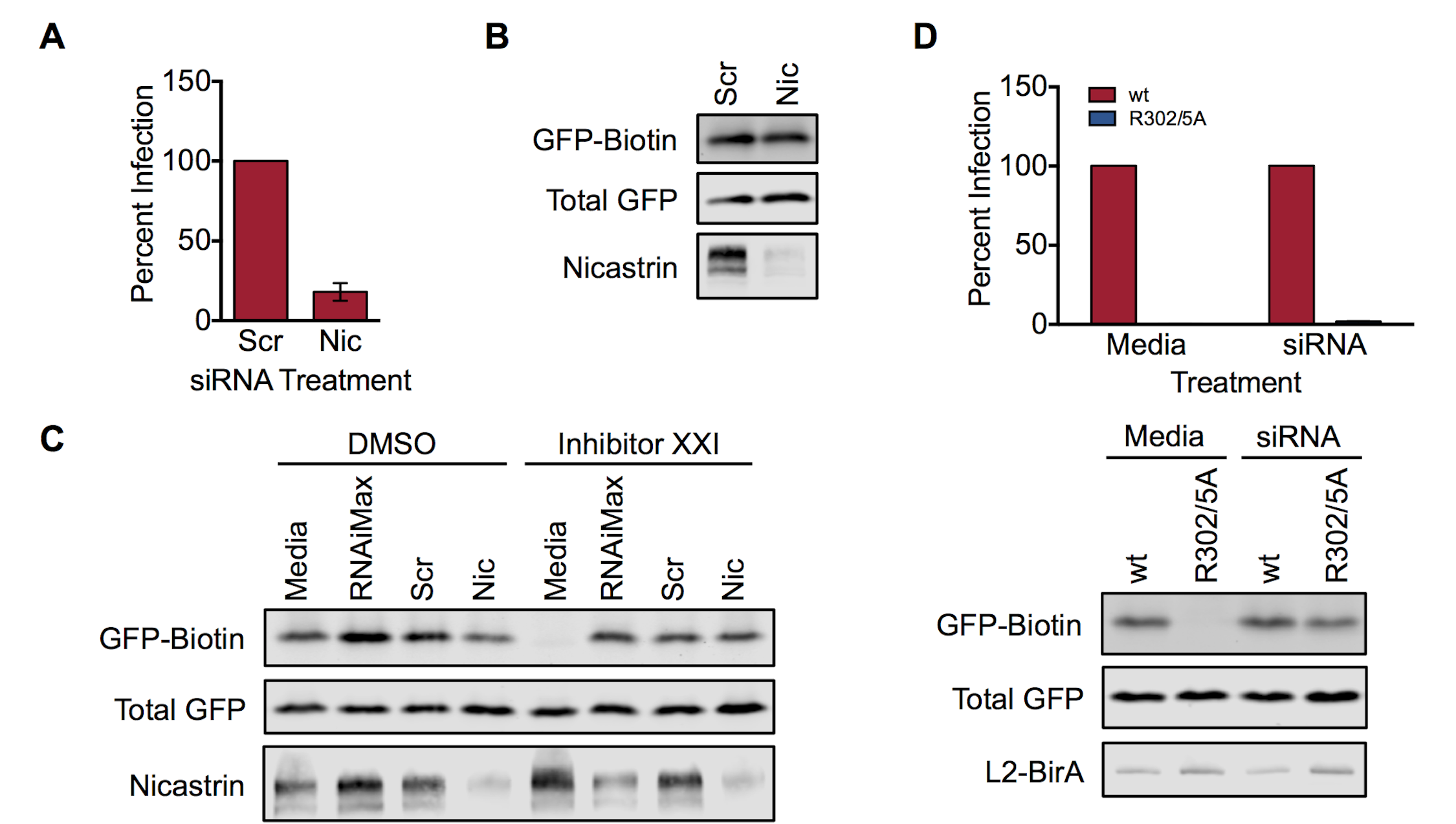

Supplement: S2 Fig — (A) Infection and (B) translocation in HaCaT GFP-BAP cells that were transfected with scramble (scr) or nicastrin (nic) specific siRNA for 24 hours and then infected with wt L2-BirA for an additional 24 hours. Infection values represent mean percent infection (±SEM, n = 2), normalized to GAPDH and expressed relative to scramble-treated cells, which are set at 100%. (C) Translocation in HaCaT GFP-BAP cells treated with media, the transfection reagent RNAiMax alone, or RNAiMax-conjugated siRNAs in the presence of the vehicle DMSO or γ-secretase inhibitor XXI. (D) Infection and translocation in HaCaT GFP-BAP treated with media or scramble siRNA for 24 hours and then infected with wt L2-BirA or R302/5-BirA for an additional 24 hours. Infection values represent mean percent infection (±SEM, n = 2), normalized to GAPDH and expressed relative to the wt sample for each condition, which are set at 100%. (TIFF) [file ppat.1006200.s002.tiff]

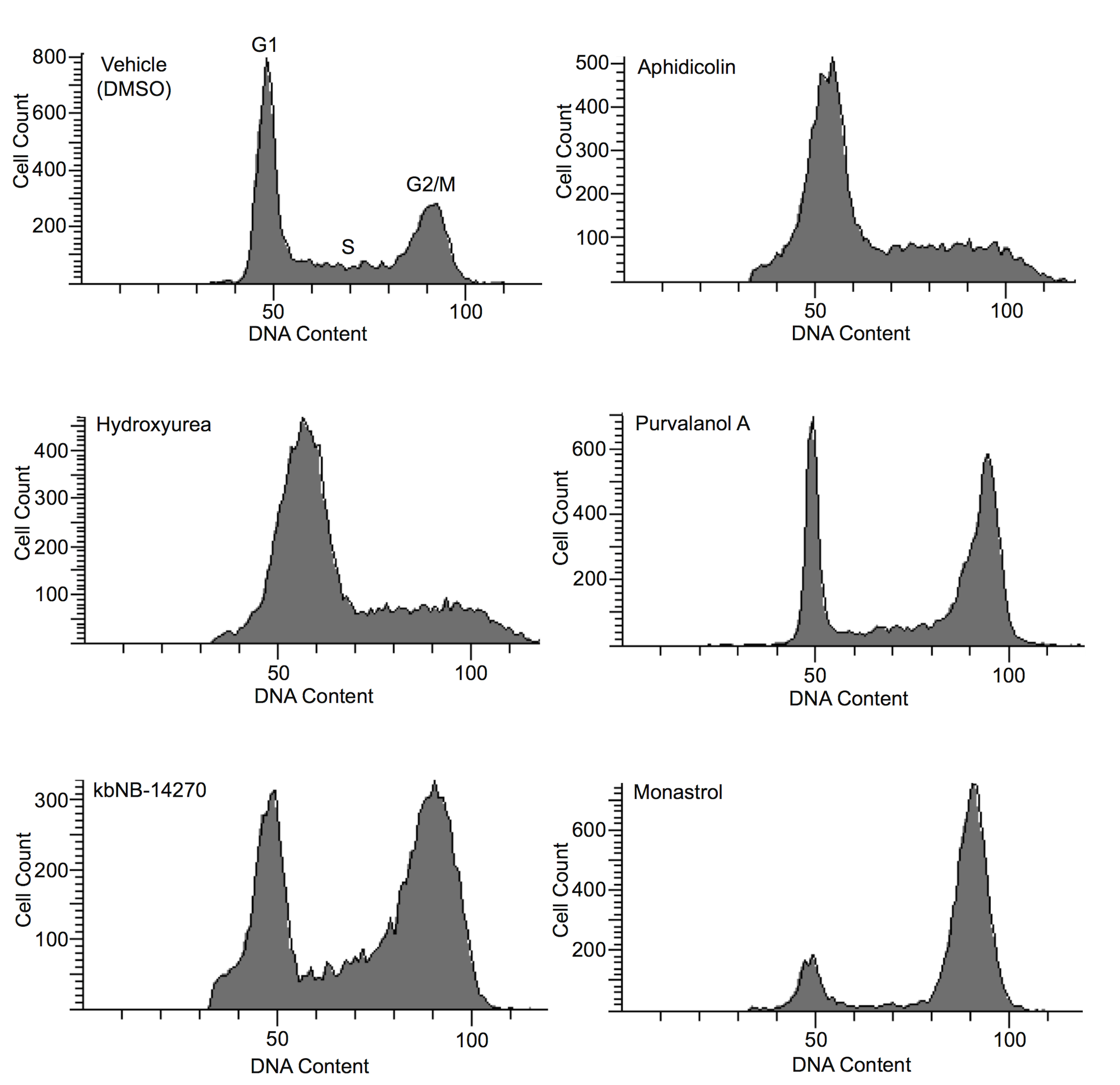

Supplement: S3 Fig — Flow cytometry of HaCaT GFP-BAP cells treated with various cell cycle inhibitors or vehicle control for 24 hours, fixed and analyzed for DNA content by propidium iodide. G1, S, and G2/M peaks are indicated on the vehicle (DMSO) profile. (TIFF) [file ppat.1006200.s003.tiff]

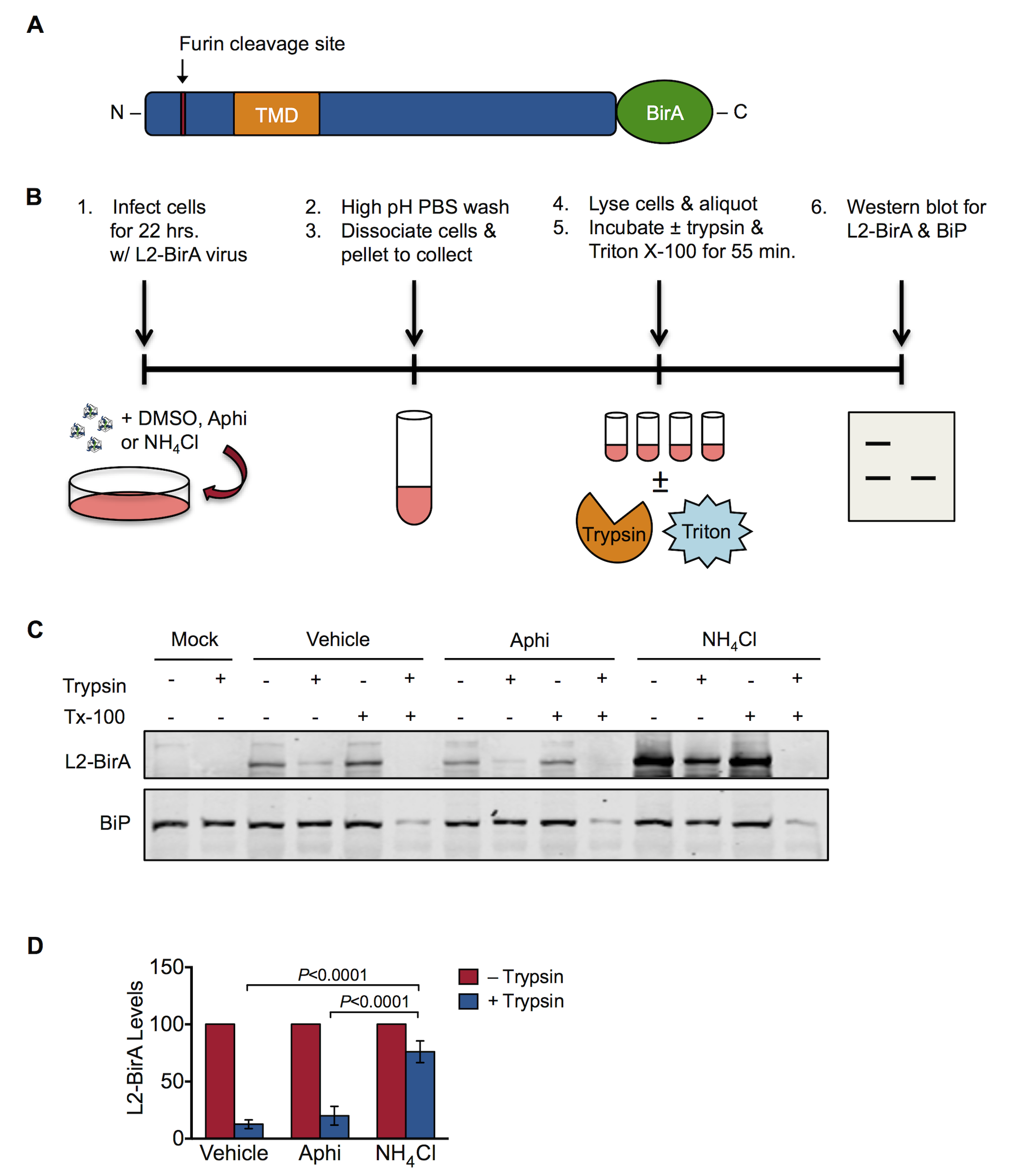

Supplement: S4 Fig — (A) Diagram of L2-BirA fusion protein showing furin cleavage site and transmembrane domain (TMD). (B) Diagram of the trypsin digestion assay experimental setup. Briefly, HaCaT GFP-BAP cells were infected with L2-BirA PsV for 22 hours in the presence of DMSO, Aphi, or NH4Cl. Cells were then washed with alkaline PBS and trypsinized to remove extracellular virus and lift the cells from the dish. Cells were gently pelleted and lysed by shearing. Crude lysate was aliquoted equally among four tubes for treatment ± trypsin and TX-100, and then incubated for 55 minutes at 37°C prior to processing for SDS-PAGE and western blot. (C) Anti-BirA and anti-BiP stains of infected cell lysates, treated as indicated. (D) Densitometry values represent mean L2-BirA levels, normalized to total BiP and expressed relative to the -trypsin condition for vehicle, Aphi and NH4Cl (±SEM, n = 3). (TIFF) [file ppat.1006200.s004.tiff]

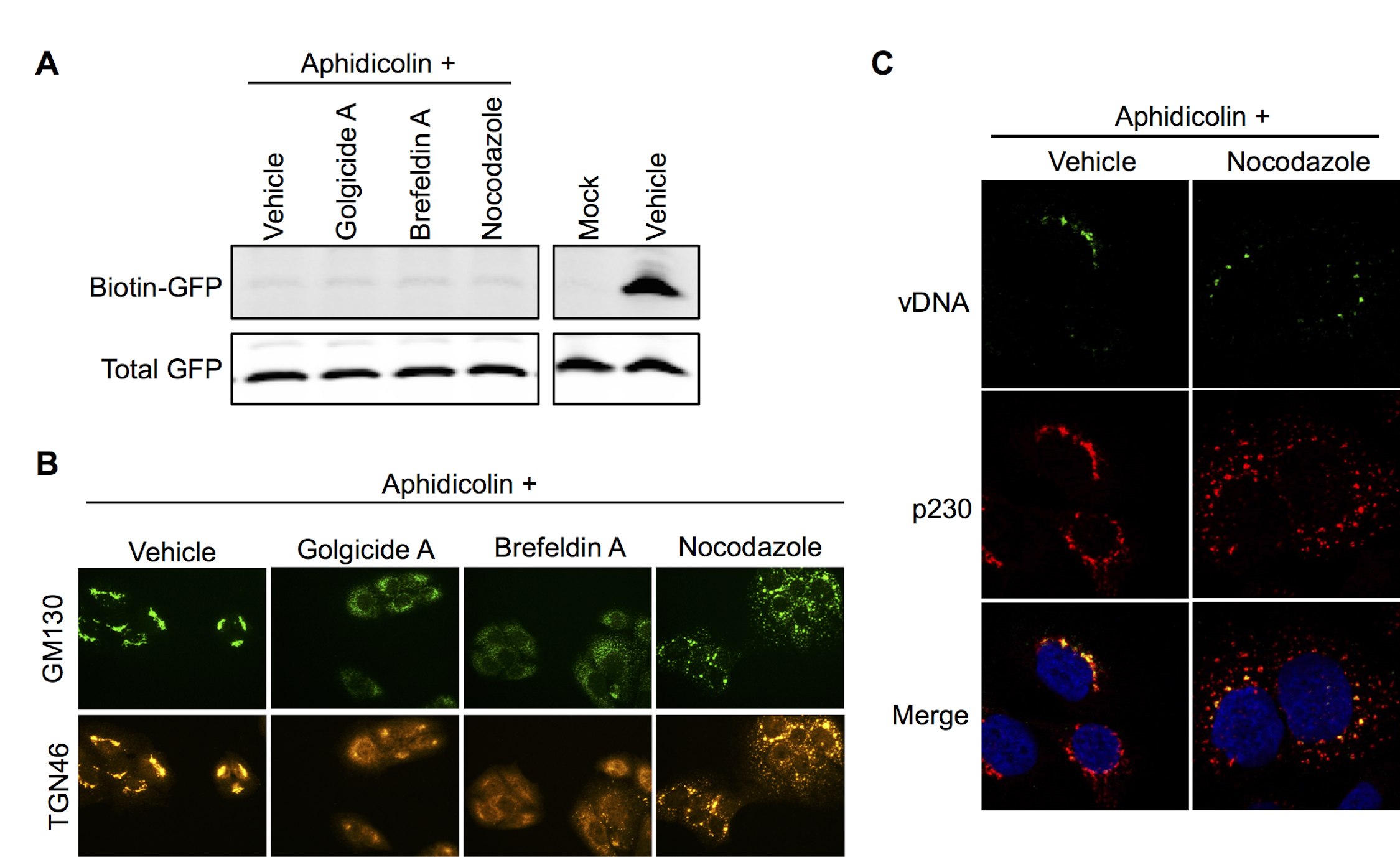

Supplement: S5 Fig — (A) Representative translocation blot of HaCaT GFP-BAP cells infected in the presence of aphidicolin for 24 hours and then treated with aphidicolin plus GDDs for 4 additional hours. (B) Representative epifluorescent images of HaCaT cells treated with aphidicolin for 24 hours and then treated with aphidicolin plus GDDs for an additional 4 hours. Cells were stained with anti-GM130 (green, cis-Golgi marker) and TGN46 (red, trans-Golgi marker). (C) Representative slices (0.35 μm) of HaCaT cells infected with wt PsV containing EdU-labeled DNA in the presence of aphidicolin for 30 hours, then exposed to aphidicolin plus nocodozole for 4 hours. After fixation the cells were stained with Alexa Fluor 488 azide to visualize vDNA (green), anti-p230 for the TGN (red), and DAPI to visualize nuclei (blue). (TIFF) [file ppat.1006200.s005.tiff]

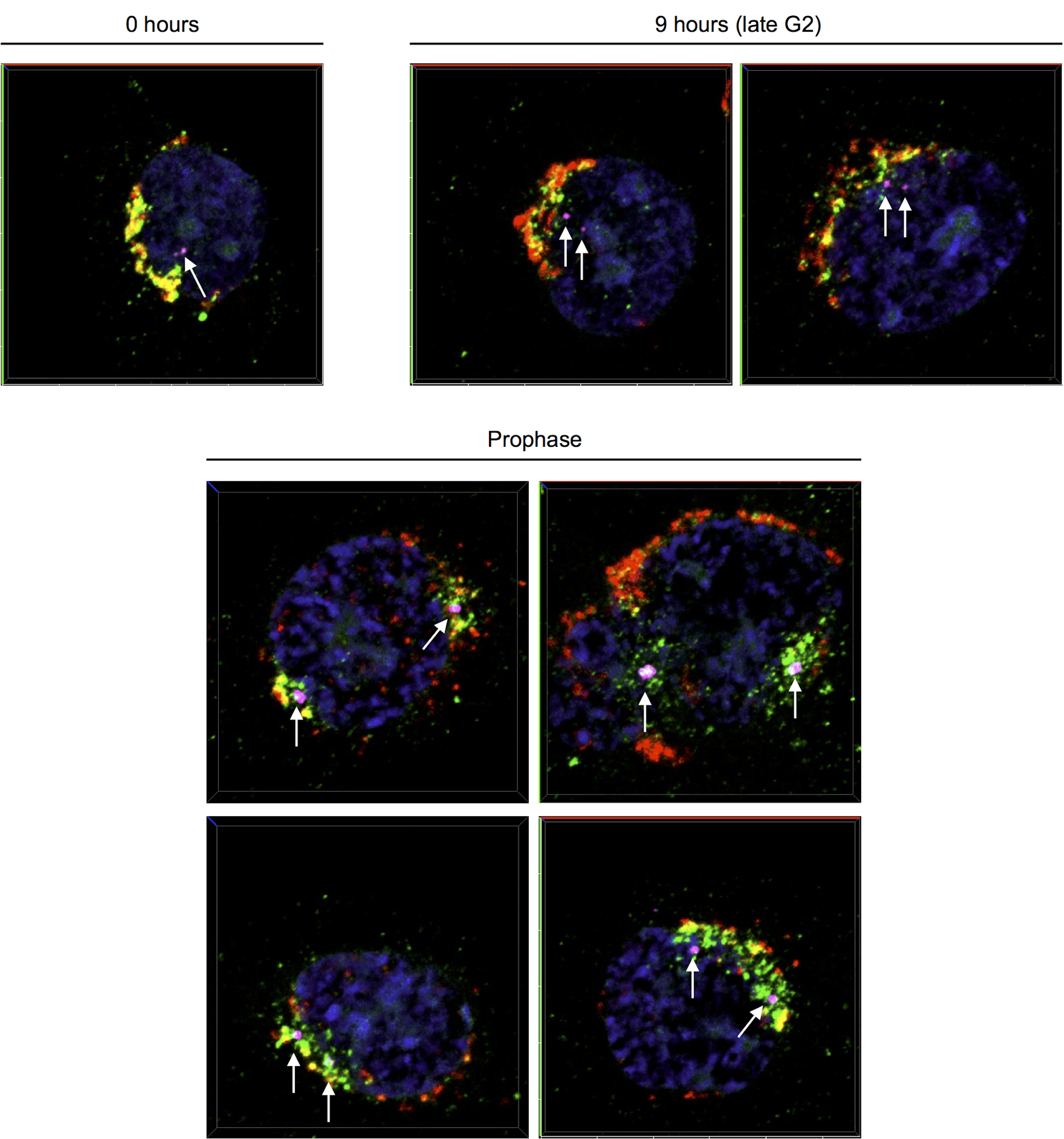

Supplement: S6 Fig — Maximum intensity projections of HaCaT cells infected with wt PsV containing EdU-labeled DNA in the presence of aphidicolin and processed as described in Fig 8. Representative images of interphase cells (0 hours post-release) and late G2 and prophase (9 hours post-release) are shown. White arrows indicate location of pericentrin. (TIFF) [file ppat.1006200.s006.tiff]

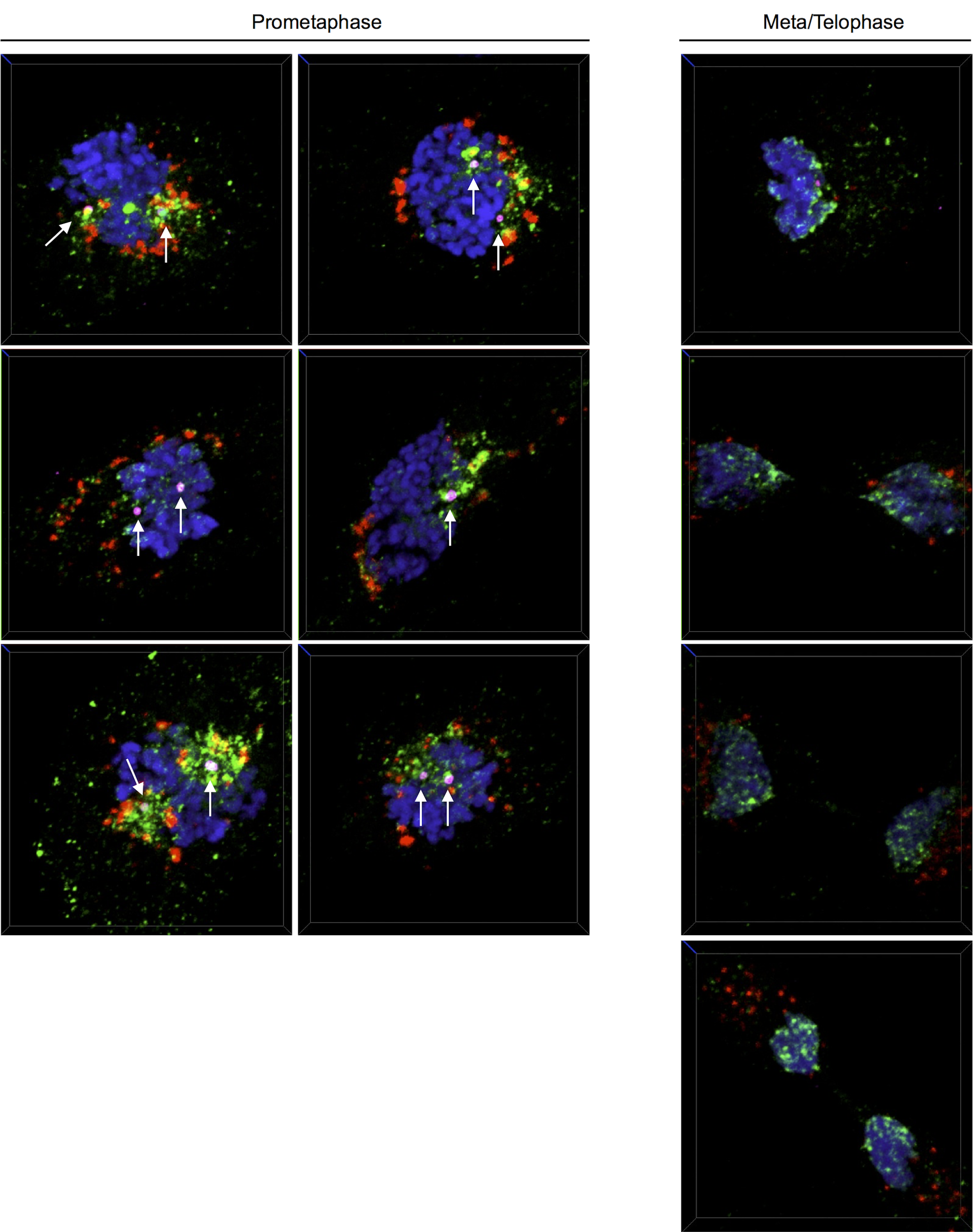

Supplement: S7 Fig — Maximum intensity projections of HaCaT cells infected with wt PsV containing EdU-labeled DNA in the presence of aphidicolin and processed as described in Fig 8. Representative images of prometaphase and meta/telophase cells (9 hours post-release) are shown. White arrows indicate location of pericentrin. (TIF) [file ppat.1006200.s007.tif]
